# Chemically Fueled, Active Droplets Prevent the Aging of Peptides into Amyloid-Like Fibers

**DOI:** 10.1021/jacs.5c12831

**Published:** 2025-11-06

**Authors:** Monika Wenisch, Michele Stasi, Simone M. Poprawa, Brigitte A. K. Kriebisch, Job Boekhoven

**Affiliations:** † Department of Bioscience, School of Natural Sciences, 9184 Technical University of Munich, Lichtenbergstrasse 4, Garching 85748, Germany; ‡ Department of Chemistry, Molecular Science Research Hub, Imperial College London, 82 Wood Lane, London W12 0BZ, U.K.

## Abstract

Protein aggregation is a hallmark of molecular aging and is implicated in various neurodegenerative diseases. Aggregation proceeds via autocatalytic, thermodynamically favored pathways. Yet in living systems, dynamic, active regulation and compartmentalization—such as in biomolecular condensates—can suppress or delay such irreversible assembly. Here, we describe a peptide that exhibits pathway-dependent self-assembly into either amyloid-like fibers or fuel-driven droplets. The peptide was designed to undergo chemical activation via a carbodiimide-driven reaction cycle, which transiently neutralizes its overall charge and promotes droplet formation. In the absence of fuel, the peptide slowly self-assembles into stable fibers through an autocatalytic process resembling amyloid aging. However, upon repeated or continuous fueling, the peptide forms active droplets that persist for days and remain resistant to fiber formation. Thus, we demonstrate that the fuel-driven active state can completely suppress fiber nucleation and growth. These findings demonstrate that the constant turnover of peptides through activation and deactivation can act as a kinetic sink, sequestering peptides and delaying the transition to the thermodynamically favored fiber state. Our results establish a minimal, chemically controlled system in which phase behavior and aging can be modulated by energy input. This work provides new insight into how nonequilibrium processes can temporally regulate self-assembly, mimicking cellular strategies for protein homeostasis. More broadly, it offers a model for studying the prevention of pathological aggregation and opens routes toward designing synthetic systems that emulate the dynamic regulation of living matter.

## Graphical abstract

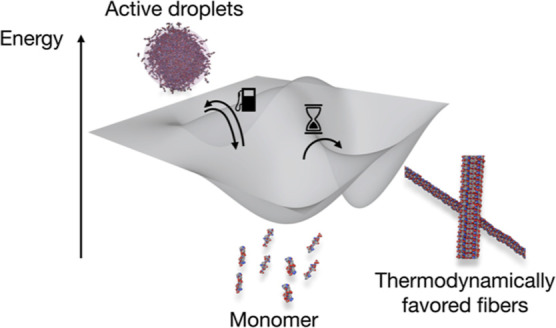

## Introduction

Aging at a molecular level refers to the accumulation of molecular damage and changes in cellular function that occur through various mechanisms, such as oxidation, genomic mutation, and aggregation of misfolded proteins.^1–3^ Aging through the aggregation of peptides is the focus of this study. While numerous mechanisms at play reverse this type of aging, these can fail. For example, chaperones can bind the hydrophobic domains of exposed proteins to prevent their aggregation or initiate their degradation.^4–6^ When the mechanisms that prevent protein aggregation fail, the consequences can be disastrous, as exemplified by the misfolding and agglomeration of tau protein associated with Alzheimer’s disease or α-synuclein aggregates associated with Parkinson’s disease.^7,8^ The process is often irreversible, and a system requires active mechanisms to prevent aging. What makes matters worse is that the aggregation of the peptides can follow an autocatalytic mechanism in which the fiber recruits new proteins to aggregate.^9–11^

In contrast to the aggregation of peptides, peptides can also assemble into a mesoscopic phase without order—liquid droplets that contain large amounts of water. These droplets have been extensively studied in the past decade in three parallel subfields: biomolecular condensates, complex coacervates, and simple coacervates. Biomolecular condensates are dynamic membraneless compartments that form in the cell through liquid–liquid phase separation of proteins, nucleic acids, or their combinations.^12–14^ As they comprise high concentrations of proteins, they have been associated with accelerating protein aggregation.^13,15–19^ Besides, the liquid droplets themselves can also age and become increasingly solid with time.^20^ Thus, biomolecular condensates can be viewed as a metastable state between soluble proteins and their thermodynamically favored aggregated state. Conversely, protein droplets have also been shown to decrease the likelihood of protein aggregation by sequestering protoaggregates.^21,22^ Thus, biomolecular condensates have been demonstrated to both accelerate and inhibit the molecular mechanisms of aging.

To better understand the complexity of protein aggregation in a biological context, supramolecular systems have been developed that mimic aspects of fibrillation^9,23–25^ and protein condensation. For example, synthetic systems have been explored in which two oppositely charged poly ions combine to form liquid, polyion-rich droplets.^26,27^ These so-called complex coacervates primarily form through electrostatic interactions between two oppositely charged polyanions, leading to their complexation.^28^ They are studied to serve as molecular reaction hubs,^29,30^ protocells,^31–34^ models for membraneless organelles (MLOs),^35,36^ and other applications. In contrast to complex coacervates, simple coacervates consist of only a single poly ion that self-associates to form liquid droplets. Similarly, these droplets are studied to control chemical reactions and as protocell models.^37,38^

The formation of simple coacervates can be coupled to a chemical reaction cycle that modifies the structure of a peptide, such that its interactions are sufficiently strong to drive coacervation only in the modified state. A biological example is the self-assembly of the tau protein into coacervate droplets after its phosphorylation.^39^ Another example involves the use of pH to control the protonation state of a peptide and, consequently, the formation of simple coacervates.^40^ Peptide-based simple coacervates can promote the organization of peptides into fibers.^41,42^ Moreover, fibrillation of peptides has been demonstrated to be inhibited by the presence of droplets.^22^ While these studies are excellent models for elucidating the intricacies of droplet involvement in protein aggregation, they have predominantly focused on droplets in or near thermodynamic equilibrium, contrasting the active environment of the cell in which biomolecular condensates are frequently regulated through nonequilibrium post-translational modifications.^43^ For example, several organelles are regulated during cell mitosis through dynamic protein phosphorylation,^44^ while others are kept in a dynamic state by arginine acetylation.^45^ These modifications maintain droplets in a dynamic state, where proteins are transiently activated or deactivated, facilitating phase separation. Such active droplets possess unique properties,^46–48^ including the ability to control their size or spontaneously divide.^49,50^ However, their ability to inhibit or accelerate the aging of molecular systems remains largely unexplored.

This work describes how active droplets can prevent the aging of a peptide into fibers. We found a peptide that is initially well-soluble but slowly ages to form amyloid-like fibers through an autocatalytic, thermodynamically driven process. Excitingly, the process could be inhibited if the peptide is assembled in active, fuel-driven droplets. Like biomolecular condensates driven by dynamic post-translational modifications, our peptide is also transiently activated to form droplets when fuel is supplied and can be sustained for several days without any sign of aging into the thermodynamically favored fibers. When fuel is removed, however, the droplets dissolve, and the fibers appear. Taken together, the constant activation and deactivation of peptides to form dynamic droplets can suppress peptide aggregation into fibers. Our findings have implications for our understanding of how nonequilibrium processes can prevent the aggregation of molecules.

## Results and Discussion

### Peptide Design

We designed a short peptide, Ac-(FY­(OMe)­RCG)_2_D-OH, to form coacervate droplets driven by a chemically fueled reaction cycle. We used alternating charged amino acids—the cationic arginine (R) and anionic cysteic acid (C)—to promote ion–ion interactions between peptides. Aromatic residues, such as phenylalanine (F) and methylated tyrosine (Y­(OMe)), were added to enhance π-cation interactions, particularly between tyrosine and arginine, which is known to drive phase separation, for example, in the FUS protein.^51,52^ We used glycine (G) as a flexible spacer between the repeating units, facilitating droplet formation. Finally, we used a C-terminal aspartic acid (D) to render the peptide responsive to our previously described carbodiimide-driven reaction cycle.^26,53–60^ Specifically, the C-terminal aspartate is converted into its corresponding intramolecular anhydride by reaction with a carbodiimide as fuel (peptide activation). The activation eliminates two negative charges, thereby changing the net peptide charge from −2 to 0 (Figure 1A). We reasoned that this charge neutralization reduces electrostatic repulsion between the peptides, triggering self-association leading to the formation of simple coacervate droplets. As the anhydride hydrolyzes in aqueous solutions, the precursor is regenerated (deactivation), leading to weakened self-interaction and disassembly from the droplets (Figure 1B). Thus, the active state of the peptide is transient and confined to the droplet phase. In contrast, the deactivated peptide is expected to remain in the surrounding solution. To our surprise, we found that the peptide forms active, simple coacervates upon fuel addition, as intended, but also assembles into amyloid-like fibers over time in the absence of fuel (Figure 1C).

**Figure 1:**
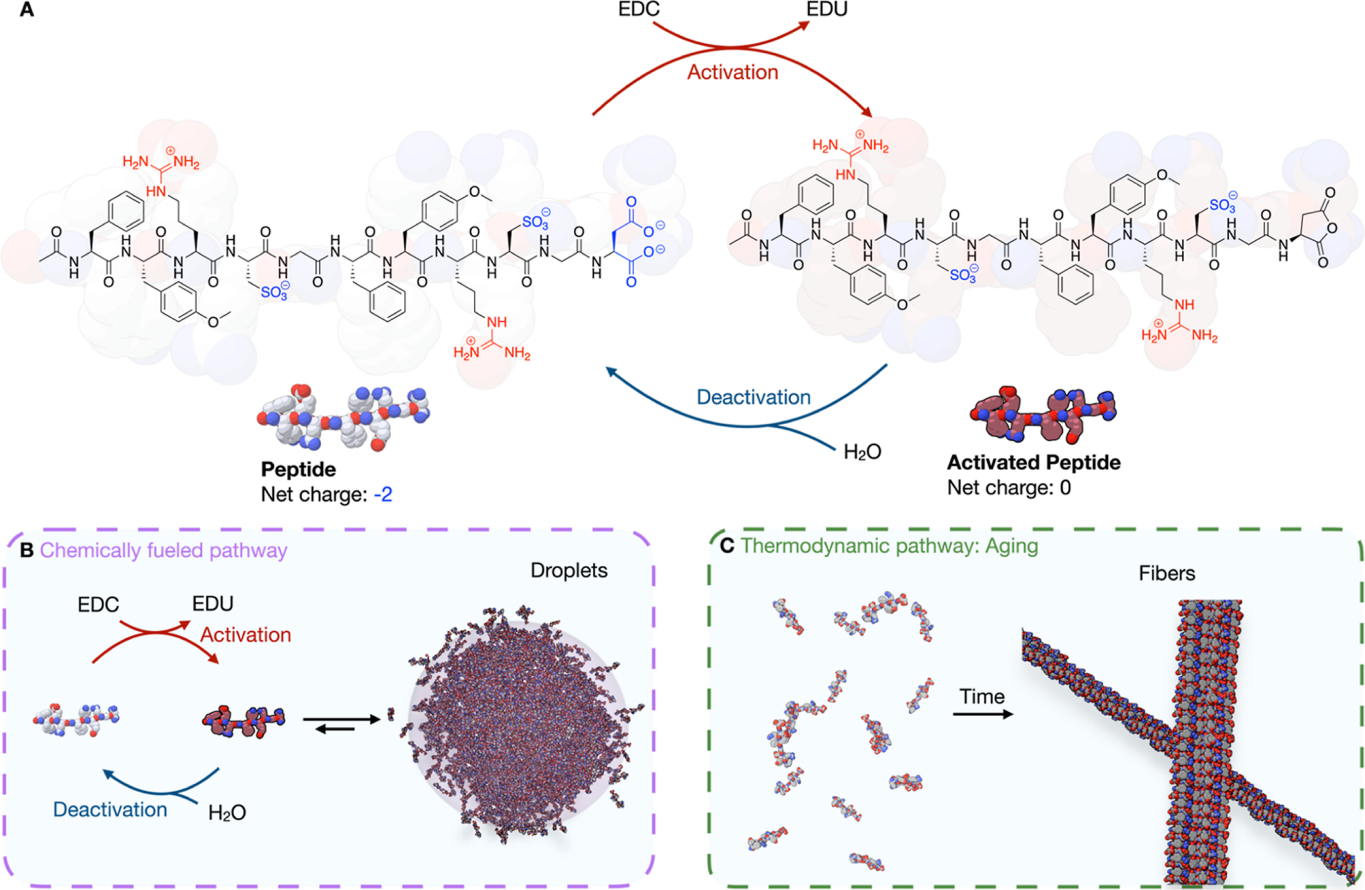
Pathway-dependent peptide assembly. (**A**) Molecular structure of the peptide sequence and the used carbodiimide-driven reaction cycle. (**B**) The chemically fueled pathway leads to dynamic, fuel-driven peptide activation and deactivation, which results in active, simple coacervate droplets. (**C**) The thermodynamic pathway converts a solution of peptide into thermodynamically favored fibers.

This observation unveils two distinct assembly pathways. The pathway to fibers is a thermodynamic pathway in which the peptide gradually forms stable fibers over time. The second pathway is driven by the conversion of carbodiimide as fuel, leading to the transient formation of active, simple coacervate droplets (Figure 1B). Once the fuel is depleted, the coacervates dissolve, and the system eventually progresses toward the thermodynamically favored fibrous state (Figure 1C).

### Aging of Peptides into Fibers

First, we characterized the thermodynamic pathway of the peptide assembly into fibers, i.e., the aging of the peptide solution. Specifically, we measured the Thioflavin T (ThT) fluorescence, indicative of amyloid-like fiber formation, as a function of time of a sample containing 5.0 mM Ac-(FY­(OMe)­RCG)_2_D-OH in 200 mM MES buffer (pH of 5.3), and 2.5 μM ThT (Figure 2A). After a lag phase of roughly 18 h, the ThT fluorescence intensity increased rapidly and plateaued after a few hours. The lag phase is the time until the first evidence of assembly is observed. Such a sigmoidal evolution of the ThT signal indicates an autocatalytic nucleation and growth process, further indications of amyloid-like behavior.^61–63^ Repeating the experiment with Nile Red yielded similar results, indicating some hydrophobic domains are formed during the assembly (Supporting Figure S1).^64^

**Figure 2:**
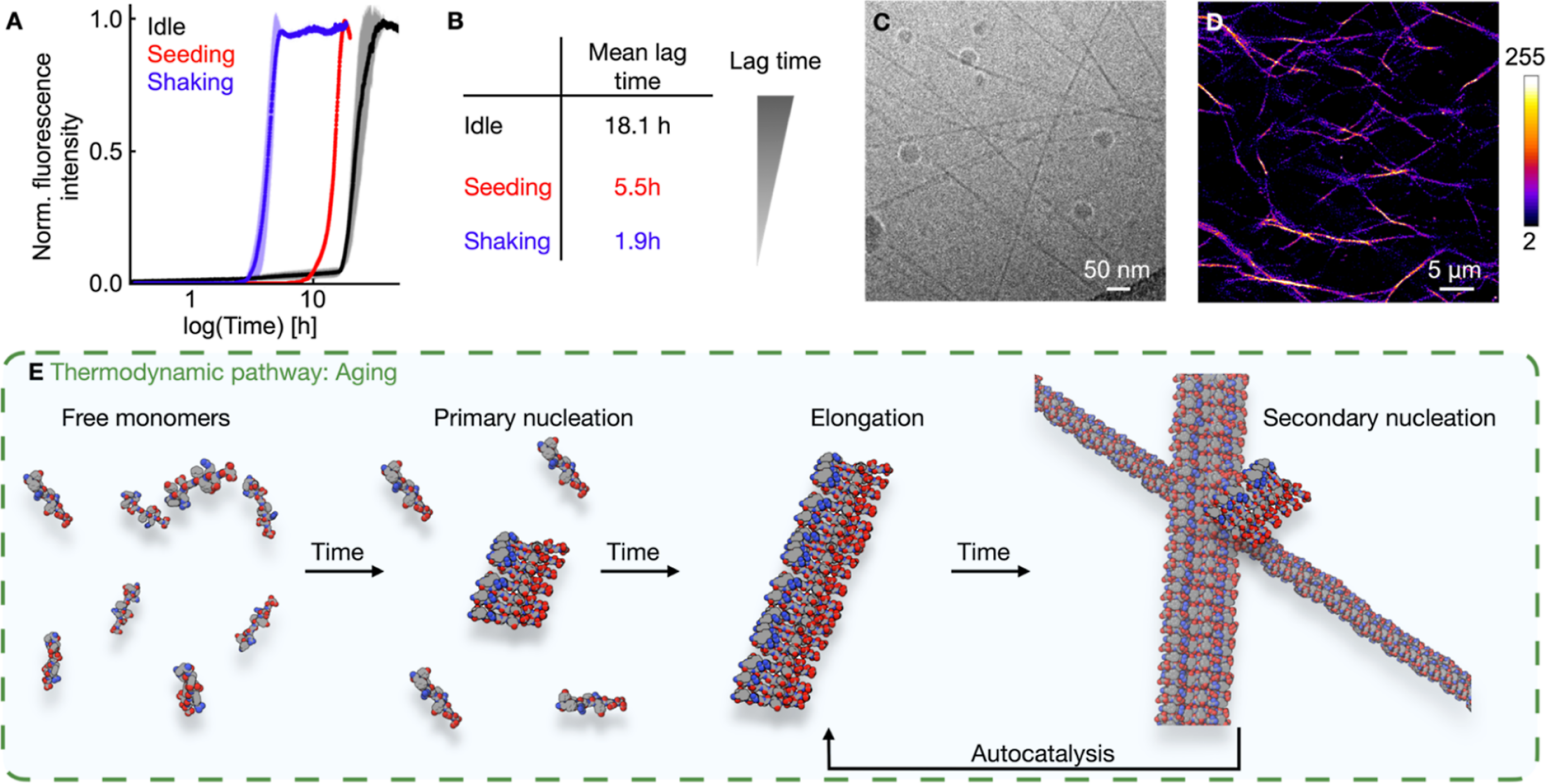
Thermodynamic pathway of the peptide self-assembly into fibers. (**A**) Kinetic studies on the fiber formation using 5.0 mM Ac-(FY­(OMe)­RCG)_2_D-OH in 200 mM MES buffer at a pH of 5.3 and 2.5 μM ThT. The normalized fluorescence intensity over time is shown for an idle (black), seeded (red), and shaken (blue) sample. Error bars are from triplicates (*N* = 3). (**B**) Comparison of the measured lag phases in B. (**C**) Cryo-TEM image of fibers in a sample of 5.0 mM Ac-(FY­(OMe)­RCG)_2_D-OH in 200 mM MES buffer at a pH of 5.3 after shaking overnight. (**D**) Confocal micrograph of fibers using the standard conditions (5.0 mM Ac-(FY­(OMe)­RCG)_2_D-OH in 200 mM MES buffer at a pH of 5.3) stained with 2.5 μM Nile Red. (**E**) Schematic representation of the involved steps of peptide fiber formation with the primary and secondary nucleation, elongation, and fragmentation.

To understand the driving forces for fiber formation, we monitored it in the presence of sodium chloride, urea, and guanidine. The addition of sodium chloride and guanidine could reduce the lag phase to less than 1.5 h (Supporting Figure S2), which we attribute to the shielding of the negative charges at the C-terminal aspartic acids, facilitating fiber nucleation. In contrast, urea showed a roughly similar lag time to the idle sample, but the slope of the signal increase was lower (Supporting Figure S2), suggesting that H-bonds are critical for fiber elongation. We have synthesized a second peptide lacking one phenylalanine (Ac-FY­(OMe)­RCGY­(OMe)­RCGD-OH), which did not form fibers, highlighting the crucial role of π-interactions in fiber formation (Supporting Figure S3).

To further corroborate the autocatalytic nature, we investigated the effect of agitation and seeding on fiber formation.^65^ Adding preformed fibers reduced the lag phase to 5.5 h (Figure 2A). Further increasing the concentration of the seeds decreased the lag phase (Supporting Figure S4). Moreover, shaking the samples between fluorescence readings further shortened the lag phase to 1.9 h (Figure 2A). Overall, shaking reduced the lag phase by ∼9.5-fold compared to idle samples, and ∼3.3-fold compared to seeded samples (Figure 2B). To confirm fiber formation, we incubated a 5.0 mM peptide solution overnight in a shaker in MES buffer (pH of 5.3) without any fluorescent dye to rule out that ThT is promoting the fiber formation.^66^

Cryogenic transmission electron microscopy (cryo-TEM) revealed long, thin fibers with an average diameter of 4.92 ± 0.39 nm (Figure 2C). The diameter is consistent with the theoretical peptide backbone length of 4.75 nm, suggesting stacked peptide monomers—likely in an antiparallel orientation as predicted by AlphaFold and supported by ATR FT-IR measurements (Supporting Figures S5 and S6). Finally, fiber formation was observed using confocal microscopy with Nile Red as the dye (Figure 2D). We explain our findings of autocatalytic fiber growth as follows. The dissolved peptide monomer can spontaneously form nucleation sites, so-called primary nucleation sites, followed by elongation (Figure 2E).^63,67^ However, this is a slow process and the rate-determining step. The autocatalytic growth from there is explained by secondary nucleation, which occurs at the surface of existing fibers. Here, peptide monomers become preorganized, facilitating the formation of new nucleation sites.^63,68^ In this way, the number of available fiber ends for elongation is increased drastically.

A preliminary fit of the kinetics of fibrillation is consistent with the hypothesis that secondary nucleation is essential in the system (Supporting Figure S7).^69^ Through this mechanism, seeding the sample reduces the lag phase. Agitation, on the other hand, accelerates fiber formation by a combination of two mechanisms: (1) primary nucleation occurs preferentially at interfaces (e.g., air–water or container wall-water), and agitation facilitates detachment and dispersion of these nuclei into the bulk solution;^24,70,71^ (2) the number of elongation-competent ends is increased due to the breakage of fibers and the more readily detachment of secondary nucleation sites from the fiber surface.^63,68^ Indeed, increasing interfacial area through vigorous pipetting also reduced lag phases (Supporting Figure S8), though these methods were less reproducible and introduced significant variability. The mechanism of nucleation and autocatalytic growth strongly resembles the growth of amyloid fibers associated with the aging of proteins like tau or α-synuclein.^9,63^

### The Kinetic Self-Assembly Pathway

The peptide can also undergo fuel-driven phase separation into active, simple coacervate droplets. We initiated droplet formation from a fresh solution of the peptide under the same conditions as above by adding 1-ethyl-3-(3-(dimethylamino)­propyl) (EDC), the chemical fuel. The addition of fuel almost immediately turned the solution turbid, which we used as a proxy for the presence of droplets (Figure 3A). As the peptides consumed the fuel, the droplets were short-lived, and the solution became clear within minutes. Tracing the absorbance over time enabled us to track the lifetime of the droplets. By adding a second batch of fuel, it was possible to form the active, simple coacervates again (Supporting Figure S9). With increasing EDC concentration, the lifetime of the formed simple coacervates increases (Figure 3A) in line with previous work on complex coacervates.^26,53,72,73^ By confocal microscopy, we found the formation of spherical droplets using a NBD-labeled peptide derivative as dye (Figure 3B, Supporting Figure S10 and Movie 1). Using particle tracing software, we determined the number of droplets, their volume, and the total volume of all droplets in the field of view, which correlated well with the observed absorbance traces over time (Supporting Figure S11).

**Figure 3:**
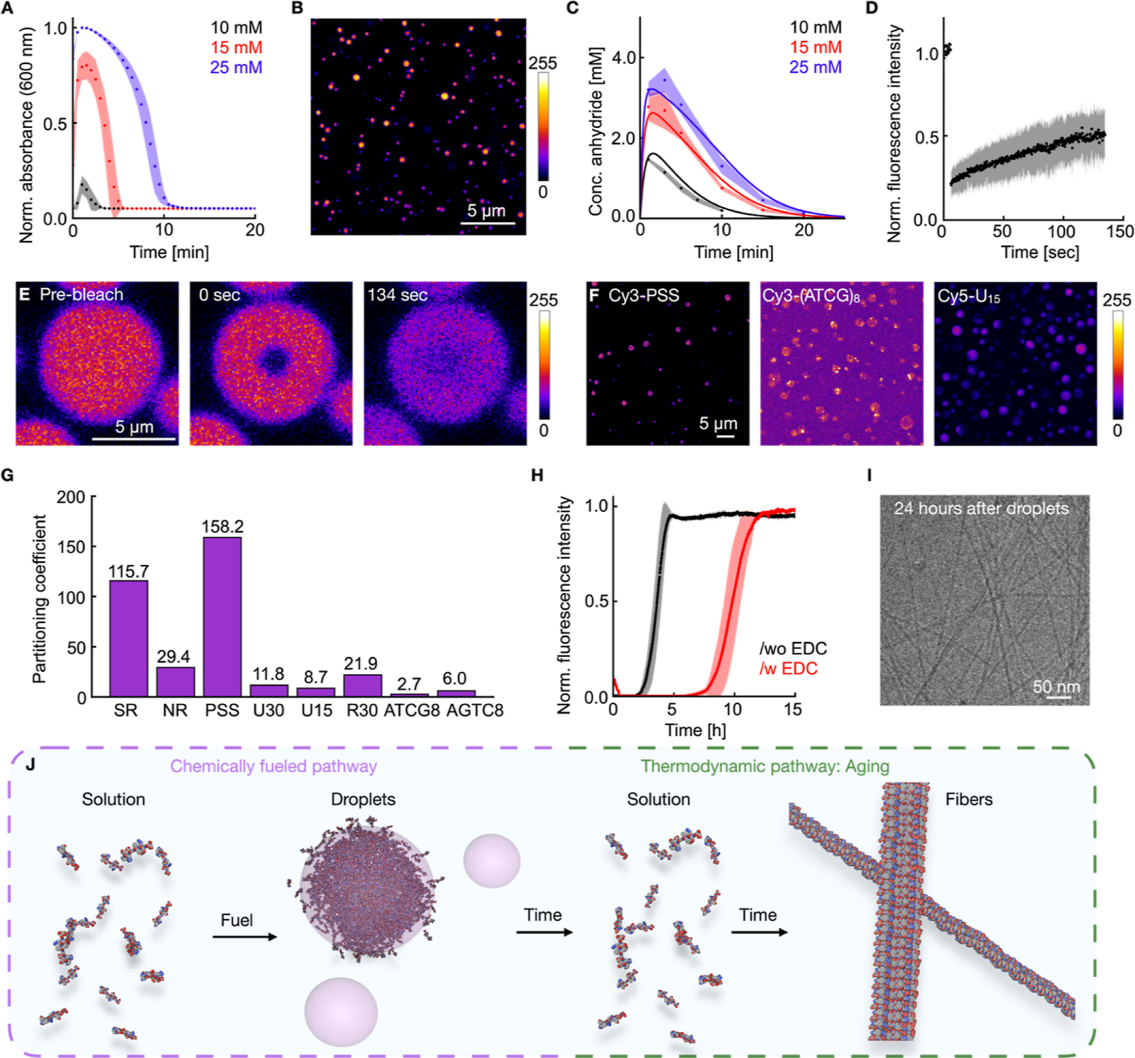
Kinetic pathway of the peptide assembly into simple coacervates. (**A**) Absorbance over time of samples containing 5.0 mM Ac-(FY­(OMe)­RCG)_2_D-OH in 200 mM MES (pH of 5.3) fueled with different concentrations of EDC. Error bars are from triplicates (*N* = 3). (**B**) Confocal micrograph of the formed active, simple coacervates using a NBD-labeled peptide derivative as dye. (**C**) The HPLC-measured anhydride concentration over time for varying EDC concentrations using 5.0 mM Ac-(FY­(OMe)­RCG)_2_D-OH in 200 mM MES (pH of 5.3). Error bars are from triplicates (*N* = 3). (**D**) Normalized fluorescence intensity over time after photobleaching an active, simple coacervate using 5.0 mM Ac-(FY­(OMe)­RCG)_2_D-OH in 200 mM MES (pH of 5.3) and 25 mM EDC using 200 nM NDB-labeled peptide as dye. Error bars are from triplicates (*N* = 3). (**E**) Confocal micrograph of the FRAP experiment shown in D. (**F**) Confocal micrographs of the partitioning experiments using 5.0 mM Ac-(FY­(OMe)­RCG)_2_D-OH in 200 mM MES (pH of 5.3) and 200 nM of the dye/labeled molecule. (**G**) Bar plot of the partitioning coefficients of the experiments shown in F. (**H**) Normalized fluorescence intensity over time using 5.0 mM Ac-(FY­(OMe)­RCG)_2_D-OH in 200 mM MES (pH of 5.3) and 2.5 μM ThT, without EDC (black) and with 25 mM EDC (red). In between the measurements, the samples were shaken. Error bars are from triplicates (*N* = 3). (**I**) Cryo-TEM image of fibers in a sample of 5.0 mM Ac-(FY­(OMe)­RCG)_2_D-OH in 200 mM MES (pH of 5.3) and 25 mM 24 h after droplet formation. (**J**) Schematic representation of the formation of simple coacervates upon chemical fuel addition to the peptide solution. After the depletion of the fuel, the coacervates dissolve, followed by the aging of the peptide to form the thermodynamically favored fibers.

To better understand the underlying kinetics of the chemical reaction cycle, we measured the anhydride and fuel concentration over time using a recently established quenching method.^74^ A kinetic model was used to describe the kinetics, and the rate concentrations were varied to fit the model to the data (Figures 3C, Supporting Information: kinetic model). These rate constants are in line with those for similar peptides published by our group^26,53^ and imply that the activated peptide has a half-life of roughly 39 s. From the kinetic model, we could determine that a minimum of 1.4 mM of the activated peptide is needed to form droplets. Removing one phenylalanine in the repeating unit (Ac-FY­(OMe)­RCGY­(OMe)­RCGD-OH) led to an increase in this critical coacervation concentration to approximately 30 mM (Supporting Figure S3), demonstrating that aromatic interactions are essential in the formation of droplets. Moreover, the addition of 850 mM of sodium chloride led to droplets with a significantly reduced lifetime, highlighting the importance of electrostatic interactions in coacervate formation. When the same amount of urea or guanidine was added, coacervate formation was inhibited entirely (Supporting Figure S12), further demonstrating that hydrogen bonds are crucial for coacervate formation.

To verify the liquid nature of the droplets, we performed fluorescence recovery after photobleaching (FRAP) experiments on the simple coacervates (Figure 3D and E, Movie 2). We derived an average diffusivity constant of 3.4 × 10^–3^ μm^2^/s from three independent experiments (Methods: FRAP and Supporting Table S1). Besides, we tested the uptake of a range of molecules in the simple coacervates, the dyes Nile Red, ThT, and Sulforhodamine B, which all partitioned into the droplets (Figures 3F and G, Supporting S13). Additionally, we tested negatively charged polyanions, such as polystyrenesulfonate (PSS), DNA, and poly uracil (PolyU), as well as positively charged polycations, namely R30 (Figure 3G). We found structured DNA barely partitioned into droplets, likely due to the limited electrostatic interactions and H-bonds.^75,76^ The interactions between the peptide and PolyU are weaker than those with PSS, resulting in a lower partition coefficient for PolyU.^77^ Since simple coacervates took up polyanions like PSS, we tested whether the peptide was also able to form complex coacervates with higher PSS concentrations. We found that with increasing PSS, the size of the resulting complex coacervates got smaller and their lifetime is shortened (Supporting Figure S14). Higher PSS concentrations required more activated peptide to form complex coacervates due to the increased electrostatic repulsion, leading to shorter lifetimes and smaller droplets. In conclusion, the peptide forms simple coacervates in response to chemical fuel. Unlike our previous work on complex coacervates, the droplets form at a significantly lower activated peptide concentration and without the need for a polyanion for assembly.

Next, we tested whether the peptide’s droplet formation affects the fiber formation, even after the fuel-dependent droplets had dissolved. We monitored the fiber formation after the peptide was activated with EDC to form active, simple coacervates by measuring the absorbance and the fluorescence intensity of ThT of a sample fueled with EDC. First, simple coacervates formed, which dissolved after roughly 20 min, evidenced by the emergence of turbidity and its disappearance with no fluorescence signal (Supporting Figure S15). Roughly 7 h after the active droplets had dissolved (Figure 3H), an increase in the fluorescent intensity of the ThT signal was observed, indicating the start of the fiber formation. At this time point, no coacervates were observed, and the fibers nucleated from solution. Compared to the samples without EDC, and thus solutions that never produced droplets, the fiber formation was delayed by 5 h, which we attribute to the presence of EDU as waste (Supporting Figure S16). Indeed, experiments in which EDU, the waste product of the reaction cycle, was added instead of EDC, so no coacervates are formed, resulted in a slower fibrilization. Despite this delay of the formation, the morphology of the fibers, which were formed after the formation and dissolution of the simple coacervates, remained unchanged, as confirmed by cryo-TEM (Figure 3I).

We conclude that the peptide can form active droplets in the presence of fuel. When the fuel is depleted, the droplet decays, regenerating the peptide, which, without further fuel addition, ages to form the thermodynamically favored fibers (Figure 3J).

### Suppression of Fiber Formation

Given that the peptide can be sequestered in the droplets, we wondered if the aging of the peptide solution into amyloid-like fibers could be suppressed indefinitely by the active droplets, provided that the droplets remained present indefinitely. To test this hypothesis, we had to constantly supply fuel to sustain the active, simple coacervate droplets for several hours, which we achieved by mixing *N*,*N*′-diisopropylcarbodiimide (DIC), a hydrophobic carbodiimide fuel, with mineral oil and topping the fueled samples with it. The DIC diffuses in the aqueous solution and activates the peptide, sustaining the active droplets. The formed *N*,*N*′-diisopropylurea (DIU) diffuses back to the mineral oil phase, eliminating the influence of the formed waste on the fiber formation.^76^ That way, droplets can be sustained for over 100 h (vide infra). We first confirmed that mineral oil itself does not inhibit fiber formation; we performed control experiments using peptide solutions topped with plain mineral oil. Due to the oil–water interface, we observed a lag phase of roughly 50 h compared to 16 h with an air–water interface, but the fibers were still formed, evidenced by a ThT assay and confocal micrographs (Figures 4A and B, Supporting S17).^70,71^

**Figure 4:**
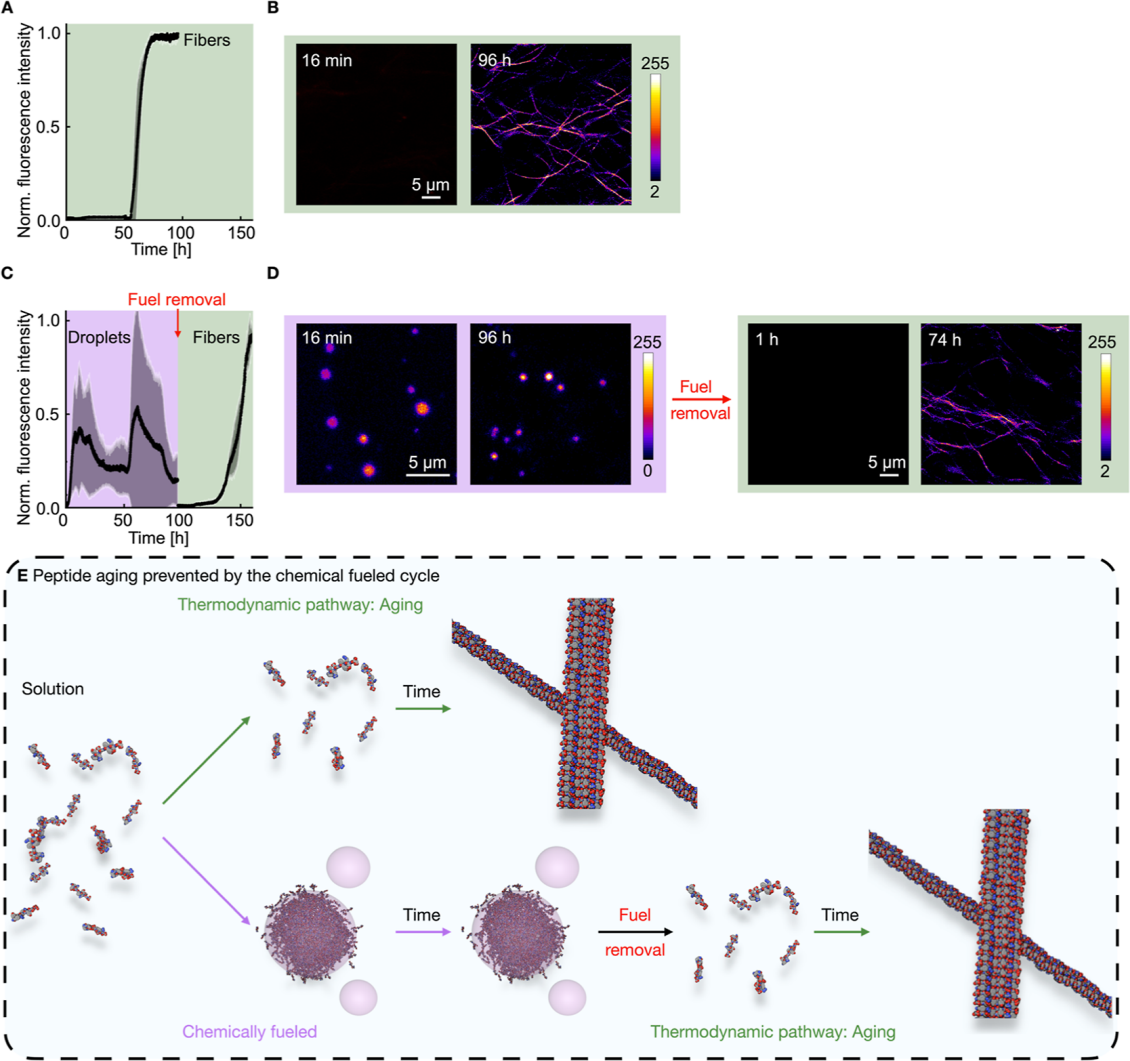
Combination of the thermodynamically driven fiber formation and chemical-fueled, active, simple coacervates. (**A**) Normalized fluorescence intensity over time of 5.0 mM Ac-(FY­(OMe)­RCG)_2_D-OH in 200 mM MES (pH of 5.3) and 2.5 μM ThT topped with mineral oil. Error bars are from triplicates (*N* = 3). (**B**) Confocal micrographs of the samples in A after different time points. (**C**) Normalized fluorescence intensity over time of 5.0 mM Ac-(FY­(OMe)­RCG)_2_D-OH in 200 mM MES (pH of 5.3), 25 mM EDC, and 2.5 μM ThT topped with DIC-loaded mineral oil. Error bars are from triplicates (*N* = 3). (**D**) Confocal micrographs of the samples described in C after different time points. (**E**) Schematic representation of the temporal control of the fiber formation using a chemical fuel.

Next, we tested whether the coacervates could be sustained for the entire duration required for fiber nucleation and growth. To sustain the active droplets for hours, we prepared the samples as previously (5.0 mM Ac-(FY­(OMe)­RCG)_2_D-OH in 200 mM MES (pH of 5.3), 2.5 μM ThT, 25 mM EDC) and topped them with mineral oil loaded with 1 M DIC. We monitored absorbance over time to check the presence of the active, simple coacervate droplets. Initially, the absorbance peaked while EDC and DIC were actively fueling the system. Then, it decreased as EDC was consumed. Sedimentation and droplet fusion resulted in a gradual increase in turbidity, which eventually stabilized (Supporting Figure S18). The DIC-fueled samples showed fluorescence intensity from the beginning since the simple coacervates take up ThT (Figure 4C). The ThT signal was noisy, however, due to scattering of the droplets. The presence of simple coacervates was confirmed at multiple times by confocal microscopy (Figure 4D). Excitingly, there was no evidence of fiber formation even after more than 90 h when the samples were continuously fueled with DIC. FRAP experiments of the droplets, which were sustained for 3 days to ensure that the droplets were still liquid-like. We found no statistically relevant difference in the diffusivity coefficient of the droplets directly after fueling and after sustaining them for 3 days (Supporting Figure S19, Supporting Table S1, and Movie 3).

To test whether the suppression of the fiber formation was the result of the active droplet or simply the formation of a steady state of activated peptide, we conducted the same experiment with a lower fuel concentration. That way, the anhydride is present, but its concentration remains below the critical coacervation concentration, so the droplets are not formed. In this regime, we found no evidence of the droplets. Moreover, we did find much faster fiber formation after as little as 3 h, in contrast to over 50 h for the nonfueled samples (Supporting Figure S20). These results indicate that the anhydrification of the C-terminus accelerated the fiber formation by decreasing ionic repulsion, provided that no droplets are formed. We hypothesize that the decreased repulsion between the peptides decreases their nucleation energy, thereby accelerating the fiber formation. Given that under these steady state conditions only a small fraction of the peptides is activated, we assume that the fibers mostly comprise peptides in the nonactivated state. We thus assume that these fibers still represent the thermodynamically favored product in the energy landscape. At a high concentration of fuel, the system can also phase separate into droplets. These droplets represent a dynamic state that prevents the anhydride or peptide from forming the thermodynamically favored fibers.

These findings demonstrate that simple coacervates can prevent the aging of peptides into fibers by forming dynamic, active coacervates.^78^ This highlights how continuous activation can preserve functional, liquid compartments over extended periods. This supports the broader idea that chemically fueled phase separation can be used to regulate material state and suppress aging-like transitions.

Finally, we tested whether the fibers started nucleating and growing after the chemical fuel was removed and the droplets dissolved. Thus, we removed the fuel by washing the DIC-fueled samples after sustaining them for 96 h to remove the DIC-loaded mineral oil and replaced it with only mineral oil. After the fuel removal, the droplets disappeared, as evidenced by confocal microscopy and the absence of intensity in the ThT assay (Figure 4D). Roughly 50 h after the fuel removal, the fluorescence intensity increased again, and we could observe fibers by confocal microscopy (Figure 4C and D), confirming that fuel removal permits the transition to the thermodynamically favored fiber state.

Interestingly, the reverse was not possible—once fibers were formed, they could not be reverted to droplets. We attempted to do so by redissolving the peptide after fiber formation was complete by adding fuel and found an increase in the absorbance, indicating that some droplets were formed (Supporting Figure S21A). By high-performance liquid chromatography (HPLC) analysis, we found that roughly the same amount of peptide is activated whether fibers are present or not (Supporting Figure S21B). Unfortunately, confocal experiments revealed that some droplets within the fiber network were formed, but no complete destruction of the fiber network was possible (Supporting Figure S21C and D). These findings suggest that the activation of the peptide is insufficient to destroy the fibers and redissolve the peptide.

The combined findings lead us to conclude that once the fibers are formed through the aging of the peptide, they cannot be destroyed by the EDC-driven active simple coacervate droplets used. This finding highlights a limitation of the system. When fiber formation occurs more quickly than the formation of simple coacervates, the system will not be able to prevent the fiber formation. The processes of anhydride formation and simple coacervate formation are relatively rapid, typically taking only a few seconds, whereas fiber formation generally takes at least several minutes to hours. This means that the kinetic limitation of the system is likely to apply to only a few specific systems.

These experiments highlight how chemical fueling enables temporal control over phase transitions (Figure 4E). By modulating fuel availability, we can delay the onset of irreversible fiber formation and maintain dynamic, liquid-like compartments, demonstrating a powerful strategy to spatiotemporally regulate assembly pathways in synthetic systems.

## Discussion

The results presented here show the complex interplay between thermodynamically and fuel-driven self-assembly in a minimal peptide system. Without chemical fuel, only the thermodynamically favored peptide fibers can form through an autocatalytic amyloid-like process. In contrast, the addition of chemical fuel dynamically activates peptides, leading to the formation of simple coacervates. Excitingly, the amyloid-like fiber formation can be suppressed by forcing the peptide to form the fuel-driven droplets instead. This suppression of the fiber formation is achieved either by (1) the constant turnover of the peptide in the chemical reaction cycle or (2) by the formation of coacervate droplets. Our experiments show that the transient activation alone is insufficient to inhibit fibrillation, and therefore, we conclude that the formation of droplets somehow inhibits it, which has been reported in the literature for static coacervate droplets.^21,22^ Our results suggest that the fuel-driven active droplets can act as kinetic sinks, temporarily sequestering peptides and buffering against fiber formation. Such behavior is reminiscent of the hypothesized role of MLOs in cells, where post-translational modifications regulate liquid-like compartments.^79,80^ The MLOs are known to regulate the availability of aggregation-prone proteins.^81,82^ The ability of our minimal system to reproduce such behavior strengthens the relevance of chemically fueled coacervation as a model for biological regulation. It also provides a simple platform to study the transition from dynamic, reversible assembly to irreversible aggregation. Therefore, the system can be used as a model for treating neurodegenerative diseases.

## Conclusion and Outlook

In this work, we explored the pathway-dependent self-assembly of a designed peptide capable of forming either amyloid-like fibers or active, simple coacervate droplets. This dual assembly behavior—governed by thermodynamic equilibrium or fuel-driven activation—highlights the intricate relationship between molecular design, phase behavior, and energy dissipation. We demonstrated that continuous energy consumption can suppress fibrillation; however, dissolving the thermodynamically favored fibers remains a challenge. Future studies could address this by designing peptides with more extensive modifications upon activation or by attaching bulkier moieties to sterically hinder fiber formation, thus promoting monomer redissolution.

## Supplementary Material











## Notes

Confocal microscope movie of the simple coacervation cycle using EDC as chemical fuel (Movie1.mp4) (MP4)
Confocal microscope movie of FRAP experiment of fresh coacervates (Movie2.mp4) (MP4)
Confocal microscope movie of FRAP experiment of 3 day-old coacervates (Movie3.mp4) (MP4)
Excel file containing all the raw data (Source_data.xlsx) (XLSX)
Materials and Methods description and additional data (Synthetic protocols and analysis; Sample preparation; HPLC analysis; kinetic model; Turbidity Measurements; Confocal Fluorescence Microscopy; FRAP procedures) (PDF) (PDF)

## Abbreviations

EDC: 1-ethyl-3-(3-(dimethylamino)­propyl)
EDU: 1-[3-(dimethylamino)­propyl]-3-ethylurea
poly-U: polyuridylic acid
DIC: *N*,*N*′-diisopropylcarbodiimide
DIU: *N*,*N*′-Diisopropylurea
ThT: Thioflavin T
NMR: nuclear magnetic resonance
TEM: transmission electron microscopy
MLO: menbraneless organells
HPLC: high-performance liquid chromatography
FRAP: fluorescence recovery after photobleaching
PSS: polystyrenesulfonate
