# Supplementary material for: Chemically Fueled, Active Droplets Prevent the Aging of Peptides into Amyloid-Like Fibers
Source: J Am Chem Soc. 2025 Nov 6;147(46):42472–81. doi: 10.1021/jacs.5c12831 (PMC12636012; doi:10.1021/jacs.5c12831)
Supplement: Supplementary file 5 [file ja5c12831_si_005.pdf]

Supplementary Information for:

Chemically fueled, active droplets prevent the aging of peptides into amyloid-like fibers

Monika Wenisch[1], Michele Stasi[2], Simone M. Poprawa[1], Brigitte A. K. Kriebisch[1], Job Boekhoven[1]

[1]Department of Bioscience, School of Natural Sciences, Technical University of Munich, Lichtenbergstrasse 4, 85748 Garching, Germany.

[2]Department of Chemistry, Imperial College London, Molecular Science Research Hub, 82 Wood Ln, London W12 0BZ, United Kingdom

Supplemental items

Supplemental methods

Materials

We purchased 1-ethyl-3-(3-dimethylaminopropyl) carbodiimide (EDC), 2(-Nmorpholino-)ethanesulfonic acid (MES) buffer, trifluoroacetic acid (TFA), N,N-Dimethyl formamide (DMF) in peptide synthesis grade, Sulforhodamine B, N,N′-Diisopropylcarbodiimid (DIC), 4-cyano-4-(phenylcarbonothioylthio)pentanoic acid, Chloroform, 4,4'-Azobis(4-cyanovaleric acid and $NaBH_4$ were purchased from Sigma Aldrich. The dyes for the labeling Cyanin-5-maleimide and Cyanin-3-maleimide were purchased from Lumiprobe. All the reagents for the peptide synthesis (Fmoc-R(Pbf)-OH, Fmoc-Y(OMe)-OH, Fmoc-D(OtBu)-OH, Fmoc-F-OH, Fmoc-G-OH and Fmoc-C-OH), 1-Hydroxybenzotriazol Hydrate (HOBt), O-(1H-6-Chlorobenzotriazole-1-yl)-1,1,3,3-tetramethyluronium hexafluorophosphate (HTCU), pre-loaded Wang resign (FmocAsp(OtBu) loaded, 100-200 mesh, 0.67 mmol/g), 4-(Dimethylamino)-pyridine (DMAP), Trifluoroacetic acid (TFA,99%), Piperazine (99%), Triisopropylsilane (TIPS), N,N-Diisopropylethylamine (DIPEA), 4-Chloro-7-nitrobenzofurazan (NBD-Cl, 98%) were purchased from Sigma Aldrich and used without any further purification unless otherwise indicated. HPLC grade acetonitrile (ACN) was purchased from VWR. MilliQ-water was received from a Milli-Q® Direct 8 water purification system.

Methods

Manual solid-phase peptide synthesis under controlled heating. All peptides were synthesized on a 0.25 mmol scale. Aspartic acid preloaded Wang resin (0.5mmol,0.67mmol g−1 loading) was used for the peptide synthesis. The reaction vessel with a three-way tap was connected to a nitrogen line, and the waste flask which was connected to a water pump. The resin was swelled in dimethylformamide (DMF) with an N_2 stream for 30 min at room temperature. The peptide synthesis was performed at 68 °C. Before each coupling step, the N-terminal fluorenylmethyloxycarbonyl (Fmoc)-protecting group was cleaved using 10 mL of a 5% (w/v) solution of piperazine in DMF with 0.2M hydroxybenzotriazole (HOBt). The reaction mixture was stirred with an N_2 stream for 6 min. The deprotecting solution was drained, and the resin was washed with DMF. For each coupling, 3 eq. of amino acid were used, together with 2.8 eq. of HCTU and 6 eq. DIPEA. HCTU and DIPEA were added to the amino acid and vortexed until everything was dissolved. The coupling solution was added to the resin and stirred with an N_2 stream for 6 min. After the coupling, the solution was drained, and the resin was washed with DMF. The deprotection, washing, coupling, and washing cycle was carried out for each amino acid. After the last coupling, the peptide was acetyl end-capped at room temperature. Therefore, a final

deprotection was conducted; the resin was washed at room temperature, and 6 eq. of acetic anhydride and 6 eq. of DIPEA in DMF solution were added to the resin and stirred with N_2 stream for 10 min at RT. The resin was washed with DMF and DCM. For the NBD-labeled peptide, the acetylation step was replaced by the NBD coupling. Therefore, 3 eq. NDB, 2.8 eq. of HCTU and 6 eq. DIPEA was dissolved in DMF and added to the resin. After the coupling step, the resin was washed with DMF. Both peptides variants were cleaved and purified the in the same way. A cleavage solution consisting of 2.5% MQ-water, 2.5% triisopropyl silane (TIPS), and 95% trifluoroacetic acid (TFA) was prepared to cleave the peptide from the resin. It was added to the resin and agitated for two hours at RT. The cleavage solution was collected by filtration, and the resin was washed with dichloromethane (DCM). The solvents were removed by codistillation under reduced pressure using a rotary evaporator (Hei-VAP Core, VWR). The crude peptides were dissolved in H_2O:ACN (70:30) and purified using a reversed-phase preparative HPLC (Agilent 1260 Infinity II setup, Agilent InfinityLab ZORBAX SB-C18 column 250mmÅ~21.2mm, 5 µm particle size) with a linear gradient of H_2O:ACN each with 0.1% TFA. All peptides were lyophilized (Christ Freeze Dryer Alpha 2-4 LDplus, VWR) and stored at −20 °C. They were characterized by electrospray ionization mass spectrometry (ESI-MS, Thermo Scientific, LCQ Fleet ION Trap Mass Spectrometer) in positive and negative mode, analytical HPLC (Vanquish DUO HPLC system (Chromeleon software version 7.2.10 ES) with a Hypersil-Gold, reversed-phase C18 column (particle size: 3 µm, length: 100mm, ID: 2.1mm), eluted with a gradient of 0.1% TFA H_2O:ACN and NMR.

NMR:

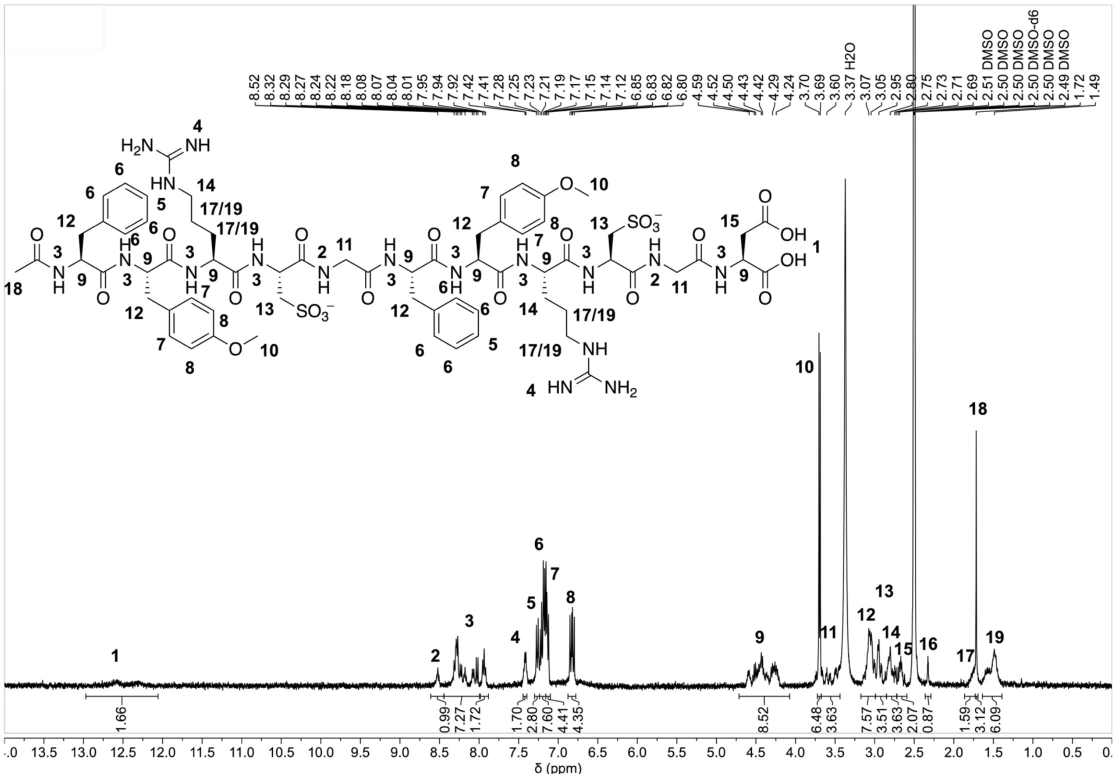

LC-MS: negative: calculated 1550.57 $[M-H]^-$ and 774.73 $[M-2H]^{2-}$, found 1550.28 $[M-H]^-$ and 775.68 $[M-2H]^{2-}$.
positive: calculated 1552.58 $[M+H]^+$ and 776.79 $[M+2H]^{2+}$, found 1553.25 $[M+H]^+$ and 777.48 $[M+2H]^{2+}$.
Retention time analytical HPLC: 6.73 min (2% to 98% ACN in MQ water, with 0.1% TFA, 12 min gradient).

Peptide synthesis Ac-FY(OMe)RCGY(OMe)RCDD-OH. The peptide was synthesized as described above. In LC-MS positive mode, we found the following masses: calculated 1405.48 $[M+H]^+$ and 703.26 $[M+2H]^{2+}$, found 1404.98 $[M+H]^+$ and 703.74 $[M+2H]^{2+}$. Retention time analytical HPLC: 8.21 min (2% to 98% ACN in MQ water, with 0.1% TFA, 10 min gradient).

Synthesis of Cy5-R30. The Cy5 labeled R30 was synthesized and purified as previously described.[1]

Synthesis of low molecular Cy3-PSS: The Cy3 labeled low molecular PSS was synthesized and purified as previously described.[2]

Nuclear magnetic resonance spectroscopy (NMR). ^{1}H-NMR-spectra were recorded on a Bruker AV-400HD NMR-spectrometer at 400 MHz. All chemical shifts δ are given in parts per million (ppm) and referenced to the residual proton signal of the respective dimethylsulfoxide-d_6 solvent (δ = 2.50 ppm). The NMR spectra were analyzed using the MestReNova software.

Electrospray ionization-mass spectrometry (ESI-MS). An LCQ Fleet Ion Trap Mass Spectrometer (Thermo Scientific) was used for ESI-MS experiments. The data was evaluated using the Thermo Xcalibur Qual Browser 2.2 SP1.48 software. The fractions of the preparative HPLC were collected, and 2 µL was injected directly into the loop.

Sample preparation. The standard concentrations for the experiments were set to 5 mM of the peptide $Ac(FY(OMe)RCG)_2D$-OH in 200 mM MES buffer at pH 5.3. For the fueling experiments, 25 mM EDC was used if not stated otherwise. All stock solutions and samples were prepared at room temperature (which was ~29°C, during summer). The peptide stock solutions (6 mM) were prepared freshly before each experiment in 200 mM MES buffer at pH 5.3. For the experiments with fluorescence dyes 2.5 µM of the dye was used. The EDC and fluorescent dyes (ThT, Nile Red) stock solutions were prepared in MQ water. The concentrations of stock solutions were 400 mM for EDC, 40 µm for the dyes and 6 mM for the peptide. The buffer concentration was sufficient to keep the pH value of all samples constant

at pH 5.3 under all applied conditions and during refueling experiments. The reaction cycle of the active, simple coacervates was started by adding the desired volume of EDC stock into the samples and mixing them by pipetting. The samples for the thermodynamic pathway were started after adding the dye.

For the kinetic studies, a quench method with amylamine was used. The samples were prepared as described, and after several timepoints, 15 µL of the sample was transferred into 30 µL of amylamine.

Sample preparation for continuously fuelling. The stock solutions were prepared as described above. The samples volume was 50 µL. The samples were placed in a a 384-well (Krystall™ well plates with transparent bottom and black walls, tissue culture plates, non-treated). After adding EDC to the fueled samples, they were topped with 20 µL of mineral oil loaded with 1 M DIC. The non-fueled samples were topped with 20 µL of mineral oil. The absorbance at 600nm and the fluorescence intensity of the ThT were monitored using an excitation wavelength of 450 nm and an emission wavelength of 482 nm.

After the non-fueled samples reached the maximum fluorescence intensity, the fueled samples were washed. Therefore, the sample was transferred into a small eppie. The aqueous solution was washed three times with 30 µL of Chloroform to remove the DIC. Afterwards, the washed aqueous peptide solution was transferred into new wells and new ThT was added to monitor the fiber growth of the washed samples. New ThT had to be added, because the ThT is extracted by the Chloroform.

Cryogenic-transmission electron microscopy (Cryo-TEM). Grids were prepared as the following: Cu-grids (C-flat™ 2/1 on 400 copper mesh) were freshly glow-discharged for 90 seconds at 45 mA and 3 Å~ 10-2 mbar. The sample (preparation see above) was diluted 1:1 with 200 mM MES buffer. 5 µL of the diluted sample was transferred onto the Cu-grids and plunge frozen using a Vitrobot Mark IV (FEI, now Thermo Scientific) at the following settings: humidity of 100%, temperature of 22 °C, 30 sec wait time, −1 blot force, 2.5 sec blot time. The cryo-TEM grids were stored in liquid nitrogen until imaging on the Tecnai Spirit microscope (FEI/Thermo Fisher).

For imaging, the cryo-TEM grids were placed into a Gatan cryo-transfer-specimen holder. Images were acquired with serialEM on a TVIPS-416 CCD camera (TVIPS).

Kinetic model. We used a kinetic model to predict the evolution of the anhydride concentration over time. The used kinetic model is described in detail in our previous work.[3] The kinetic model is based on the following reactions.

Reaction 0 (k_0):

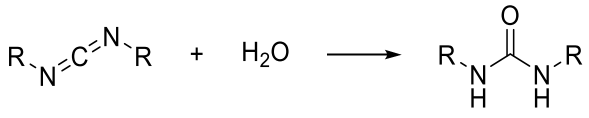

Scheme S1: Kinetic model – reaction 0 (k_0): direct hydrolysis of a carbodiimide.

The direct hydrolysis of EDC and DIC was determined in previous work with a pseudo-first-order rate constant of 1.80×10^{-4} s^{-1} and 2.90×10^{-4} s^{-1}.[4]

Reaction 1 (k_1):

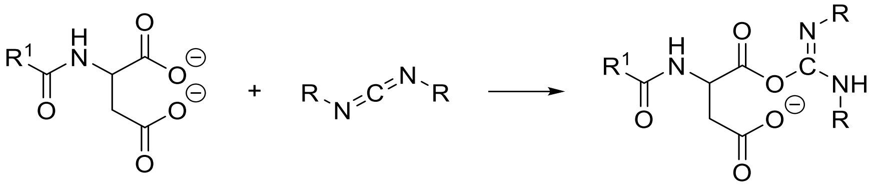

Scheme S2: Kinetic model – reaction 1 (k_1): formation of O-acylisourea by reaction of EDC with a carboxylic acid.

The second-order rate constant of the reaction of the peptide precursor with EDC or DIC, k1, was determined by monitoring the EDC consumption with HPLC and the DIC concentration by NMR.[4]

Reaction 2 (k_2):

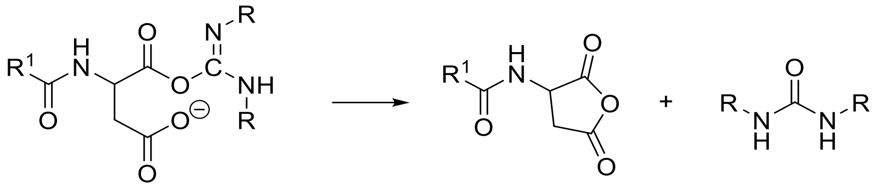

Scheme S3: Kinetic model – reaction 2 (k_2): formation of the anhydride.

The formation of anhydride and urea as waste starting from the intermediate O-acylisourea with a first-order rate constant. O-acylisourea could never be observed for the pre-precursors.

Reaction 3 (k_3):

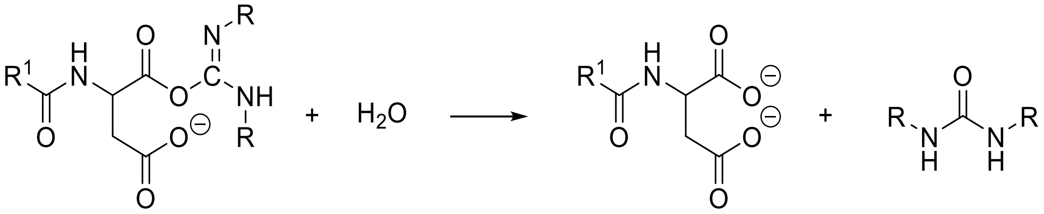

Scheme S4: Kinetic model – reaction 3 (k_3): direct hydrolysis of O-acylisourea.

Direct hydrolysis of O-acylisourea with a pseudo first-order rate constant is an unwanted side reaction. This reaction rate could not be obtained due to the not observable O-acylisourea. Thus, k_3 was fitted empirically.

Reaction 4 (k_4):

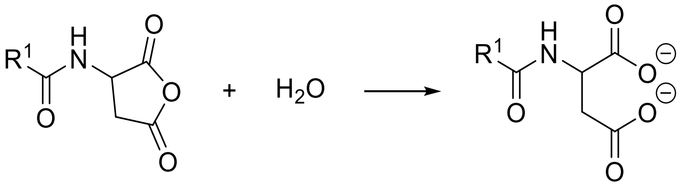

Scheme S5: Kinetic model – reaction 4 (k_4): hydrolysis of the prior formed anhydride.

The hydrolysis of the previously formed anhydride with water to the initial acid precursor takes place with a pseudo firstorder rate constant. The rate constant was fitted from the anhydride decay profile.

The kinetics of the reaction cycle were modeled according to the following set of Ordinary Differential Equations (ODEs):

$$\begin{cases} \frac{d[Ac]}{dt} = -k_{1,1}[F_1][Ac] - k_{1,2}[F_2][Ac] + k_4[An] + \alpha_1 \frac{k_{1,1}[Ac][F_1]}{\alpha_1+1} + \alpha_2 \frac{k_{1,2}[Ac][F_2]}{\alpha_2+1} \\ \frac{d[F_1]}{dt} = -k_{1,1}[Ac][F_1] - k_{0,1}[F_1] \\ \frac{d[W_1]}{dt} = +k_{1,1}[Ac][F_1] + k_{0,1}[F_1] \\ \frac{d[F_2]}{dt} = -k_{1,2}[Ac][F_2] - k_{0,2}[F_2] \\ \frac{d[W_2]}{dt} = +k_{1,2}[Ac][F_2] + k_{0,2}[F_2] \\ \frac{d[An]}{dt} = -k_4[An] + \frac{k_{1,1}[Ac][F_1]}{\alpha_1+1} + \frac{k_{1,2}[Ac][F_2]}{\alpha_2+1} \end{cases}$$

Where [Ac],[F],[An],[W] are respectively the concentration of precursor, fuel, anhydride and waste and the indices i and j are the reaction number and fuel type. To run the simulation with one fuel concentration kept constant, we imposed that $\frac{d[F_2]}{dt} = 0 \wedge \frac{d[W_2]}{dt} = 0$. The intermediate O-acylurea does not appear explicitly. We applied a quasi-steady-state approximation to the evolution of O-acylurea overtime assuming that is quickly formed and quickly consumed, therefore its concentration throughout the experiment remains low. The approximation allows us to substitute the concentration of the undetectable species in the systems of ODEs with a term depending on experimentally accessible concentrations.

$$\frac{d[I]}{dt} = +k_1 \cdot [Ac][F] - k_2[I] - k_3[I] \cong 0$$

$$[I] = +\frac{k_1 \cdot [Ac][F]}{k_2 + k_3}$$

$$with \qquad \alpha = \frac{k_3}{k_2}$$

To fit the systems of ODEs to experimental data and extract a confidence interval via bootstrap, we used the package “Kinmodel” (available at https://github.com/scotthartley/kinmodel) with a custom-written model (available at https://github.com/MWenisch/kinetic-model_SC). The package is an integration of Scipy.

Analytical high-performance liquid chromatography (HPLC). To check the purity of the used peptide stocks an analytical HPLC (ThermoFisher, Vanquish Duo UHPLC, HPLC) with a Hypersil Gold 100 2.1 mm C18 column (3 mm pore size) was used. A linear gradient (starting from 2% to 98% ACN in 12min) of H_2O: ACN, each with 0.1% TFA, was used. The measurements were monitored using a UV/VIS detector at 220, 254, and 280 nm.

UV/Vis measurements on a plate reader. UV-Vis measurements in a 96-well plate (tissue culture plates, non-treated) format were performed on a SpectraMax® ABS Plus plate reader from Molecular Devices. The absorbance at 600 nm was used as a measure for turbidity for a sample volume of 100 µL per well. The temperature was set to 25 °C.

Fluorescence plate reader measurements. The fluorescence plate reader measurements were performed in a 384-well (Krystall™ well plates with transparent bottom and black walls, tissue culture plates, non-treated) using a Varioskan LUX multimode plate reader. The absorbance at 600 nm was used as a measure for the turbidity for a sample volume of 50 µL per well. For the fluorescence intensity measurements with ThT an excitation wavelength of 450 nm and emission wavelength of 482 nm was used. The samples were prepared as described above. The temperature of the incubator was set to 25 °C.

Coating Procedure for Fluorescence Microscopy Experiments. In order to minimize the wetting of the coacervate droplets on the glass surface, a modified coating procedure of Spoelstra and coworkers was applied.[5] Micro-well plates(IBIDI, µ-Slide Angiogenesis Glass Bottom) were washed with soap water, MQ water, and ethanol. Afterward, the slide was dried with pressurized air. Then, the slide was plasma cleaned for 3 minutes followed by the addition

of 50 µL of a 5% poly(vinyl) alcohol (PVA) solution in MQ water to each well. After 15 minutes, the PVA solution was removed, and the wells were washed with MQ water and dried with pressurized air. Finally, the wells were placed into an oven at 80 °C for 30 minutes.

Fluorescence Microscopy. A Leica SP8 confocal microscope with a 63x water immersion objective was used to analyze the coacervate evolution over time in bulk using coated ibidi chambers and in the microfluidic reactors. To monitor the simple coacervates NBD-labeled peptide, which was exited at 488 nm and detected from 498 to 630 nm with a HyD detector. The fibers where stained with Nile Red, which was exited at 552 nm and detected from 560 to 700 nm with a HyD detector. The pinhole was set to 1 Airy unit. To analyze the evolution of the coacervate droplet, the entire microfluidic reactor was imaged with a time series using z-stacks with 2 µm steps between each z-plane (in total, 21 z-planes). The resolution was set to 512 × 512 pixels with a zoom of 3.5 and a scan speed of 700 Hz (bidirectional) for the coacervates. The resolution was set to 4096 × 4096 pixels with no zoom and a scan speed of 700 Hz (bidirectional) for the fibers.

Processing of the Fluorescence Microscopy images. The Leica Lighting function using the auto settings was used for the images of the fibers to obtain higher resolution. The images and time lapse of the coacervate droplets were exported without any changes. Fiji was used to adjust the brightens and contrast of the images and to include the timestamps and scale bars.

Fluorescence recovery after photobleaching (FRAP) experiments. A Leica SP8 confocal microscope with a 63x water immersion objective was used to analyze the fluorescence recovery after photobleaching the coacervates. The coacervates were stained with NBD-YRCGFYRCGD-OH. The measurements were performed in a microscopy well plate chamber (IBIDI, µ-slide Aniogenedis Glass Bottom) that was coated with PVA. The sample was bleached with a 488 nm laser and imaged at 498 to 630 nm with a PMT. The pinhole was set to 1 Airy unit, and the images were acquired at a resolution of 256 × 256 pixels with a zoom of 7.5 with a scan rate of 700 Hz. Recovery data was background and photofading corrected by Eq.(1)[6]

$$\mathrm{I_{corrected}(t)} = \frac{\mathrm{I_{raw}(t) - I_{background}(t)}}{\mathrm{I_{fading}(t) - I_{background}(t)}} \tag{1}$$

With $I_{corrected}(t)$: background and photofading corrected fluorescence intensity at time point t, $I_{raw}(t)$: raw fluorescence intensity at time point t, $I_{background}(t)$: fluorescence intensity of

background at time t, and $I_{fading}(t)$: fluorescence intensity of a neighboring not bleached droplet at time point t.

The corrected intensities were normalized by the prebleach intensity using Eq. (2)[6]

$$I_{normalized}(t) = \frac{I_{corrected}(t)}{I_{prebleach}} \tag{2}$$

With $I_{normalized}(t)$: normalized fluorescence intensity, $I_{prebleach}$: fluorescence intensity before bleaching.

For the fit of the FRAP recovery time trace Eq. (3)[7]:

$$y = a\,(1 - e^{\tau_{1/2} \times x}) + b \tag{3}$$

With *a* and *b*: fitting parameters and $\tau_{1/2>}$ recovery half-time.

The diffusivity constant was calculated using Eq. (4)[6]:

$$D = 0.224 \times \frac{r^2}{\tau_{1/2}} \tag{4}$$

With D: diffusivity constant and r: radius of the bleached spot.

The FRAP data were fitted individually to obtain the recovery half-times and derive the diffusivity coefficients. The average and the standard deviation of the diffusivity coefficient were calculated afterward.

Table S1: Average and standard deviation of the measured diffusivity constants directly after fueling and 3 days later.

	Average of diffusivity constant	Standard deviation of diffusivity constant
Freshly fueled	3.4×10^{-3} μm²/s	0.2×10^{-3} μm²/s
Fueled for 3 days	2.4×10^{-3} μm²/s	0.6×10^{-3} μm²/s

A t-test with a confident level of 0.95 of the data shown in supplementary Table S1 was performed to test for statistical relevance using the Python package ‘pingouin’ with *$p < 0.05$, **$p < 0.01$, ***$p < 0.001$, ****$p < 0.0001$; ns, not significant ($p > 0.05$). The diffusivity coefficients directly after fueling and three days later have p value of 0.114 which means they do not show significant relevance.

Attenuated Total Reflectance Fourier Transform Infrared Spectroscopy (ATR FT-IR). A Elmer Perkin Frontier FT-IR spectrometer with a Dimond ATR measurement crystal was used for the measurements. The transmittance of the samples were measured from 4000 cm^{-1} to 600 cm^{-1}, and 16 scans were conducted per measurement. For the measurement the peptide fibers were prepared as described above without using a fluorescence dye and 5 µL of the sample were transferred to the measurement crystal.

Deconvolution of the amide I band in the ATR FT-IR spectra. For the amide I band deconvolution, a 5-peak Voigt fitting approach was used. The raw infrared spectra were first converted from transmittance to absorbance and subsequently cropped to the amide I region (1600 – 1700 cm^{-1}), which is most sensitive to protein secondary structure. A linear baseline was estimated from the endpoints of the selected region and subtracted to correct for background contributions. The corrected spectra were then fitted with a model consisting of five Voigt line shapes, representing characteristic contributions of different secondary structures: β-sheets (parallel and antiparallel) (1600 – 1635 cm^{-1}), α-helix/random coil (1640 – 1655 cm and anti-parallel β-sheets (1680 – 1690 cm^{-1}).[8,9] A linear baseline term was included in the model to account for any residual drift. Nonlinear least-squares fitting was performed using the *lmfit* package, and peak areas were estimated analytically from the Voigt parameters (amplitude, sigma, and gamma). The relative contribution of each secondary structure was then quantified as the percentage of its integrated area relative to the total amide I band area.

Supporting Figures

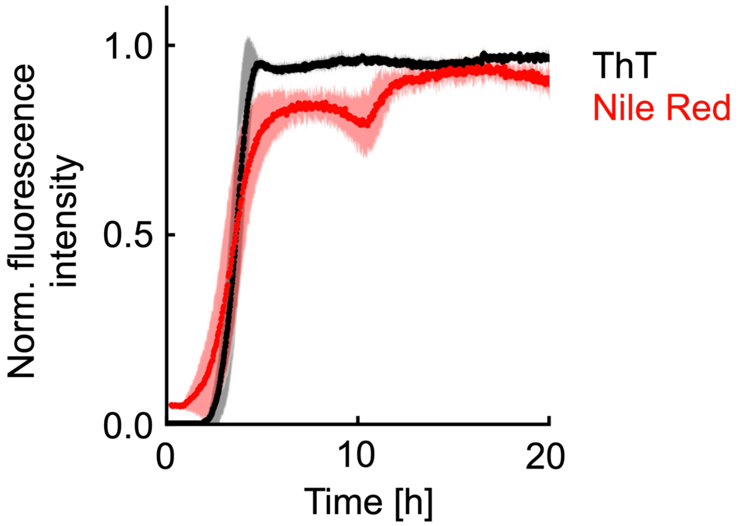

Figure S1: Normalized fluorescence intensity over time to follow the fiber formation under standard conditions (5 mM Ac-(FY(OMe)RCG)$_2$D-OH, 200 mM MES buffer at pH = 5.3, 2.5 μM fluorescent dye) using ThT (black) and Nile Red (red) as fluorescent dyes. The samples were shaken in between the measurements. Error bars are from triplicates (N = 2).

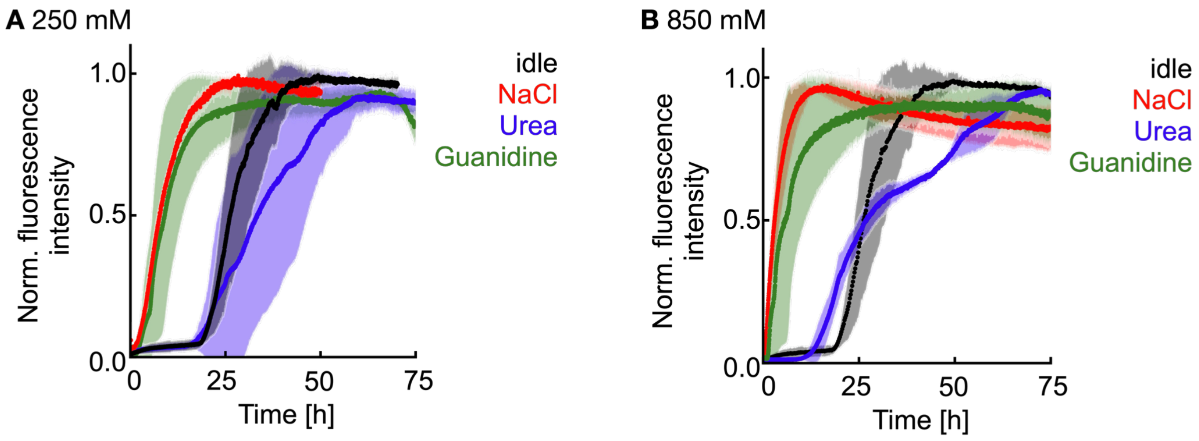

Figure S2: Fiber formation with additional salts. A) Normalized fluorescence intensity over time to follow the fiber formation of 5 mM Ac-(FY(OMe)RCG)$_2$D-OH, 200 mM MES buffer at pH = 5.3, 2.5 μM ThT and 250 mM additional salt (NaCl: red, Guanidine: green and urea: blue). Error bars are from triplicates (N = 3). **B)** Normalized fluorescence intensity over time to follow the fiber formation of 5 mM peptide, 200 mM MES buffer at pH = 5.3, 2.5 μM ThT and 850 mM additional salt (NaCl: red, Guanidine: green and urea: blue). Error bars are from triplicates (N = 3).

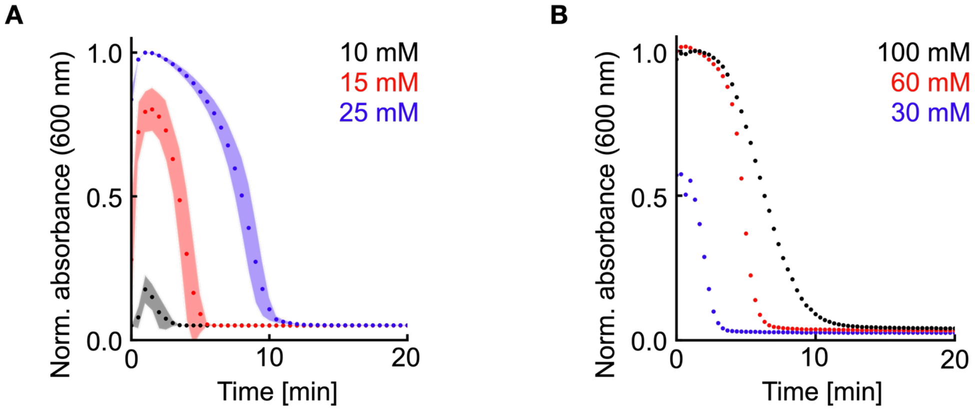

C

	Ac-(FY(OMe)RCG)$_2$D-OH	Ac-F(Y(OMe)RCG)$_2$D-OH
CCC	1.4 mM	~10 mM
fiber formation	Yes	No

Figure S3: Comparison of the two peptides Ac-(FY(OMe)RCG)2D-OH and Ac-F(Y(OMe)RCG)2D-OH. A) Normalized absorbance at 600 nm over time using 5 mM Ac-(FY(OMe)RCG)$_2$D-OH, 200 mM MES buffer at pH = 5.3 and different concentrations of EDC. Error bars are from triplicates (N = 3). **B)** Normalized absorbance at 600 nm over time using 30 mM Ac-F(Y(OMe)RCG)$_2$D-OH, 200 mM MES buffer at pH = 5.3, and different concentrations of EDC. **C)** Overview of the critical coacervation concentration of the two peptides and if fibers are formed or not.

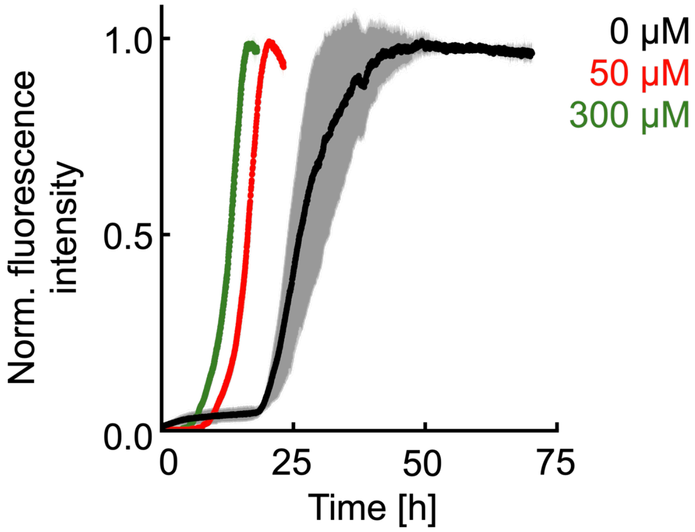

Figure S4: Normalized fluorescence intensity over time to follow the fiber formation under standard conditions (5 mM Ac-(FY(OMe)RCG)$_2$D-OH, 200 mM MES buffer at pH = 5.3, 2.5 µM ThT with different concentrations of seed, 0 µM (black), 50 µM (red) and 300 µM (green). Error bars are from triplicates (N = 3).

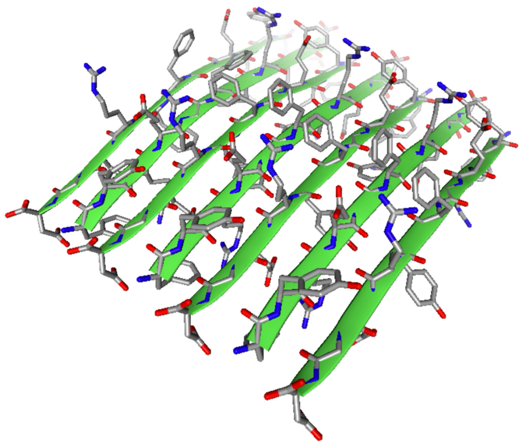

Figure S5: By AlphaFold predicted anti-parallel orientation of the used Ac-(FY(OMe)RCG)$_2$D-OH peptide in the formed fibers as the thermodynamically stable conformation of the peptide.[10]

A

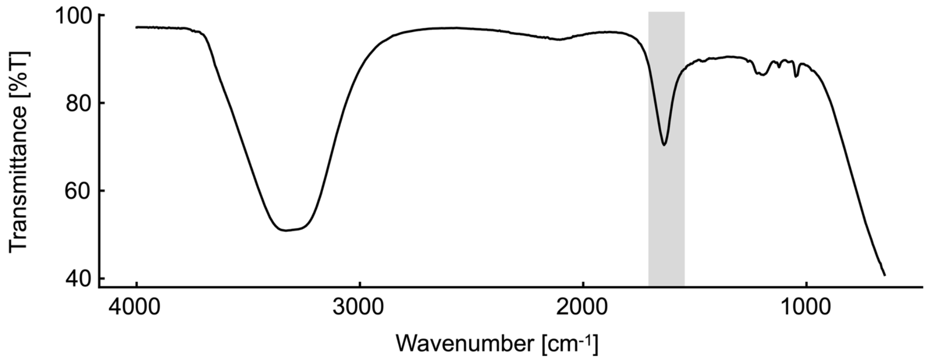

B

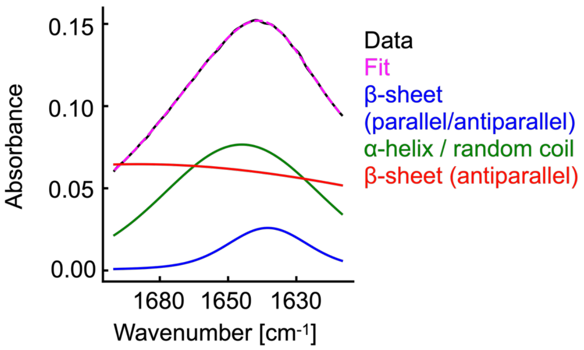

C

Type of secondary structure	Percentage
β-sheet (parallel/antiparallel)	0.9 %
α-helix / random coil	8.1 %
β-sheet (antiparallel)	91.0 %

Figure S6: Results of the ATR FT-IR measurements of the peptide fibers. A) The whole ATR FT-IR spectra of the Ac-(FY(OMe)RCG)$_2$D-OH peptide fibers. The shift in the amide I band is shaded in light gray to show the region that was used for the deconvolution. **B)** Results of the deconvolution of the amide I band of the formed peptide fibers using a five-peak fitting approach. **C)** List of the percentage contribution of the five fitted secondary structures to the amide I band in the formed peptide fibers.

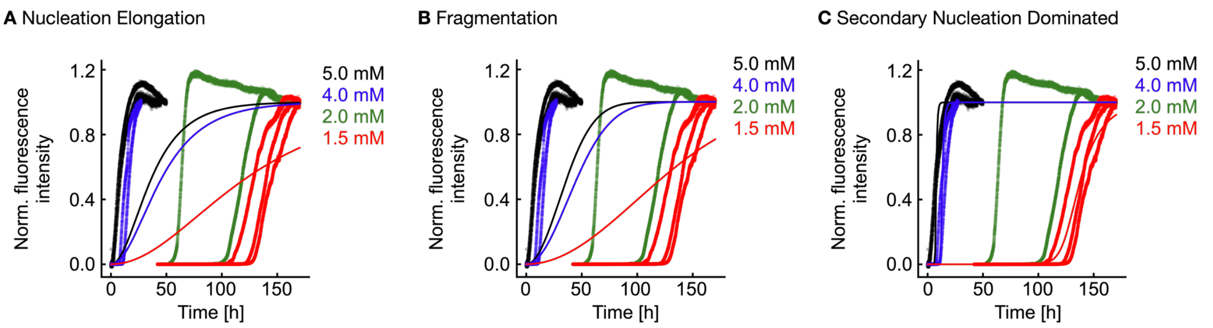

Figure S7: Kietic fitting of the fiber formation using different models. Normalized fluorescence intensity over time to follow the fiber formation using different concentrations of Ac-(FY(OMe)RCG)$_2$D-OH: 5 mM (black), 4 mM (blue), 2 mM (green) and 1.5 mM (red), in 200 mM MES buffer at pH = 5.3, 250 mM NaCl and 2.5 μM ThT. The data of 2mM peptide was not used for the fitting due to the huge data variation. The solid lines represent the kinetic fitting using different models for the fitting **A)** elongation nucleation, **B)** fragmentation and **C)** secondary nucleation.[11]

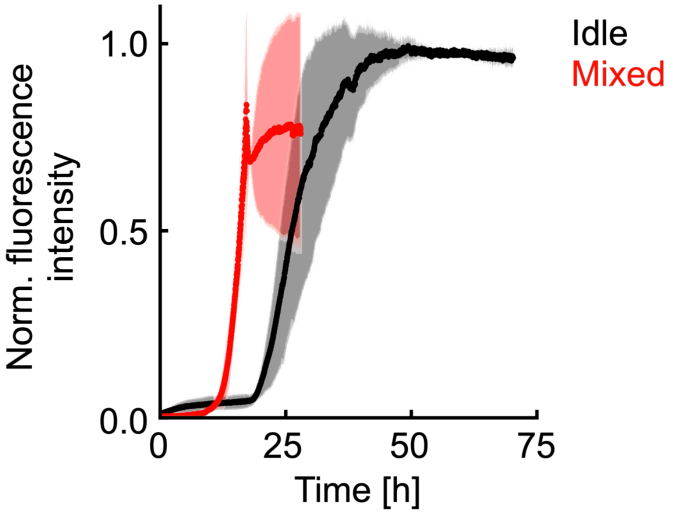

Figure S8: Normalized fluorescence intensity over time to follow the fiber formation under standard conditions (5 mM Ac-(FY(OMe)RCG)$_2$D-OH, 200 mM MES buffer at pH = 5.3, 2.5 μM ThT) under idle conditions (black) and after vigorous mixing (red). Error bars are from triplicates (N = 3).

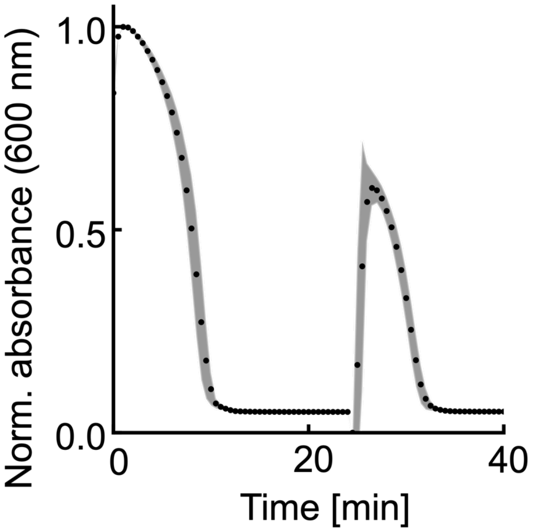

Figure S9: Normalized absorbance at 600 nm over time under standard conditions (5 mM Ac-(FY(OMe)RCG)$_2$D-OH, 200 mM MES buffer at pH = 5.3, 25 mM EDC). After 25 min another 25 mM of EDC was added to reform the coacervates. Error bars are from triplicates (N = 3).

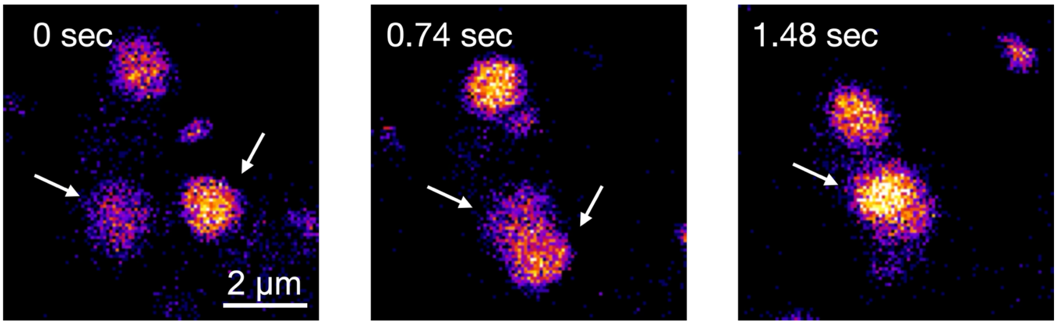

Figure S10: Time-lapse micrographs of a fusion event of active simple coacervates, roughly 3 minutes after the EDC addition. For the formation of the simple coacervates, 5 mM Ac-(FY(OMe)RCG)$_2$D-OH, 200 mM MES buffer at pH = 5.3, 25 mM EDC, and 800 nM NBD-labeled peptide were used.

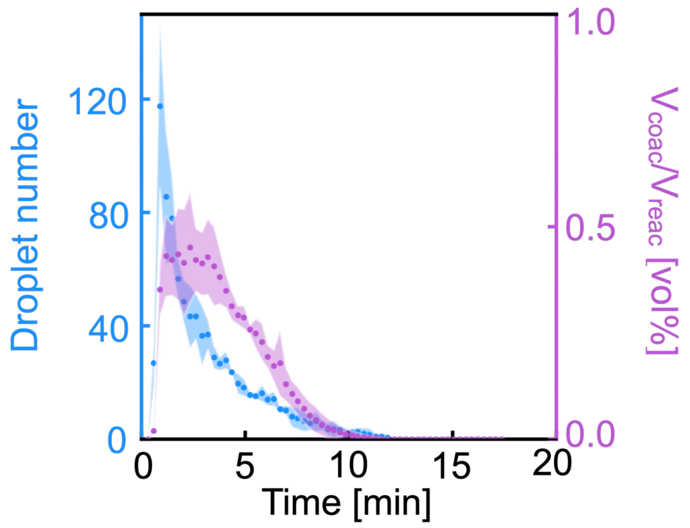

Figure S11: Droplets number over time (blue, left y-axis) and the ratio of coacervate volume vs reactor volume (violet, right y-axis) over time under standard conditions (5 mM Ac-(FY(OMe)RCG)$_2$D-OH, 200 mM MES buffer at pH = 5.3, 2.5 μM sulforhodamine B, 25 mM EDC) in microfluidic reactors. Error bars are from triplicates (N = 3).

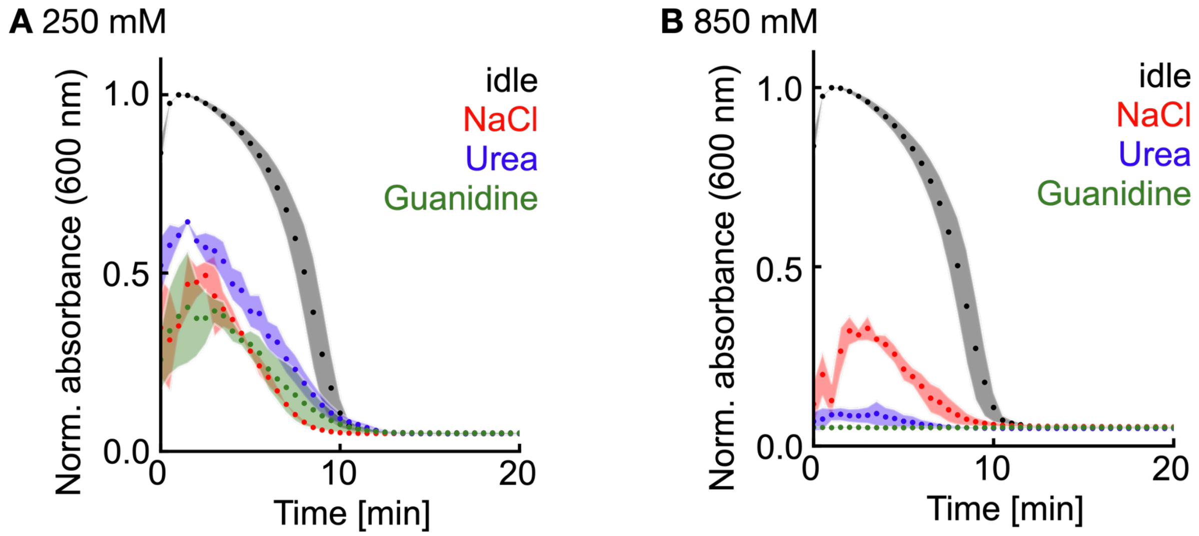

Figure S12: Coacervate formation with additional salts. A) Normalized absorbance at 600 nm over time to follow the fiber formation of 5 mM Ac-(FY(OMe)RCG)$_2$D-OH, 200 mM MES buffer at pH = 5.3, 25 mM EDC and 250 mM additional salt (NaCl: red, Guanidine: green and urea: blue. Error bars are from triplicates (N = 3). **B)** Normalized absorbance at 600 nm over time to follow the fiber formation of 5 mM peptide, 200 mM MES buffer at pH = 5.3, 25 mM EDC and 850 mM additional salt (NaCl: red, Guanidine: green and urea: blue). Error bars are from triplicates (N = 3).

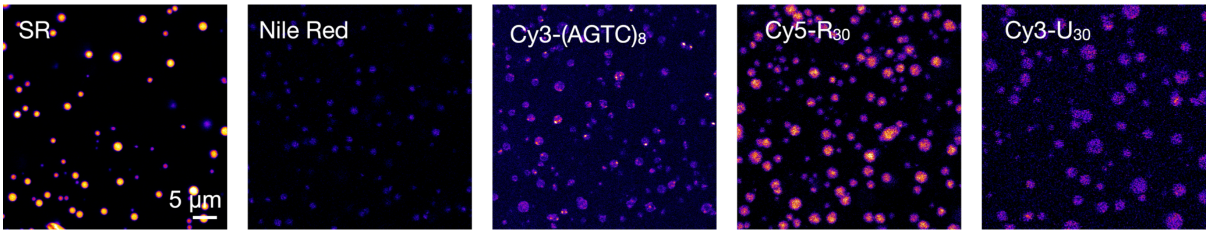

Figure S13: Confocal micrographs of partitioning experiments using standard conditions (5 mM Ac-(FY(OMe)RCG)$_2$D-OH, 200 mM MES buffer at pH = 5.3, 25 mM EDC, 200 nM fluorescent probe) testing sulforhodamineB, Nile Red, (AGTC)$_8$, Cy5-R$_{30}$ and Cy3 U$_{30}$. The scale bar is 5 µm.

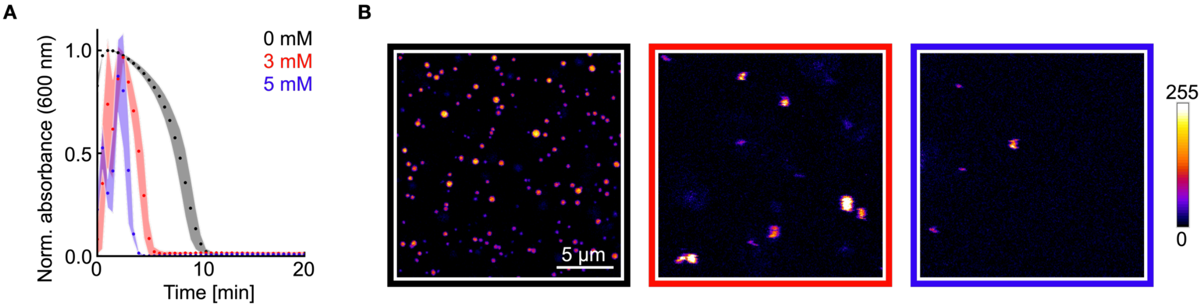

Figure S14: Formation of complex coacervates by activating the peptide in presence of PSS. A) Normalized absorbance at 600 nm over time under standard conditions (5 mM Ac-(FY(OMe)RCG)$_2$D-OH, 200 mM MES buffer at pH = 5.3, 25 mM EDC and 0 mM PSS (black), 3 mM PSS (red) and 5 mM PSS (blue). PSS with a molecular weight of 17 kDa was used for these experiments. Error bars are from triplicates (N = 3). **B)** Confocal images after roughly 2 min after the EDC addition of the conditions shown in A. The scale bar is 5 µm.

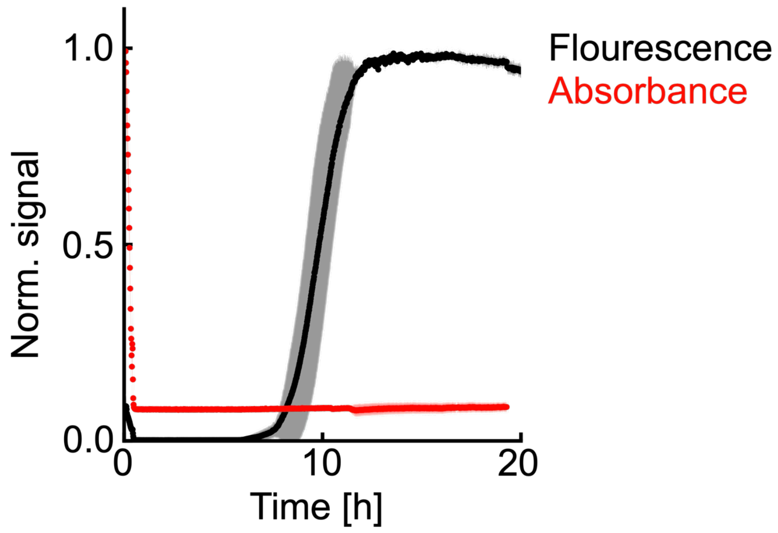

Figure S15: Normalized fluorescence intensity of ThT (black) and normalized absorbance at 600 nm over time to follow the simple coacervate formation and fiber formation using 5 mM Ac-(FY(OMe)RCG)$_2$D-OH, 200 mM MES buffer at pH = 5.3, 2.5 µM ThT and 25 mM EDC. Error bars are from triplicates (N = 3).

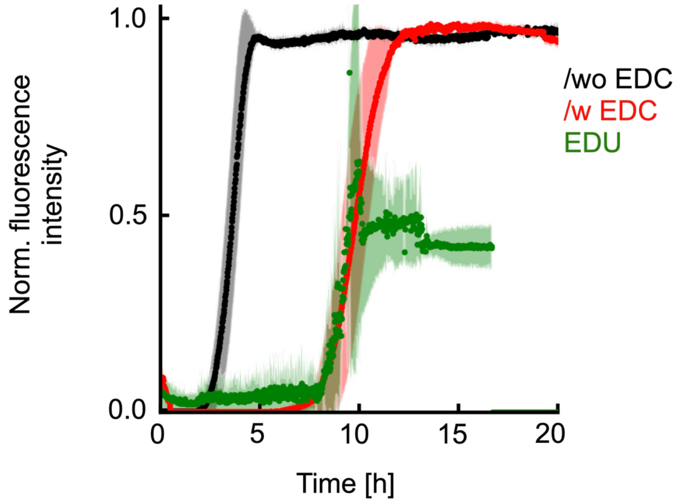

Figure S16: Normalized fluorescence intensity over time to follow the fiber formation under standard conditions (5 mM Ac-(FY(OMe)RCG)$_2$D-OH, 200 mM MES buffer at pH = 5.3, 2.5 µM ThT) under idle conditions (black), after the addition of chemical fuel (25 mM EDC, red) and the addition of 25 mM EDU (green). Error bars are from triplicates (N = 3).

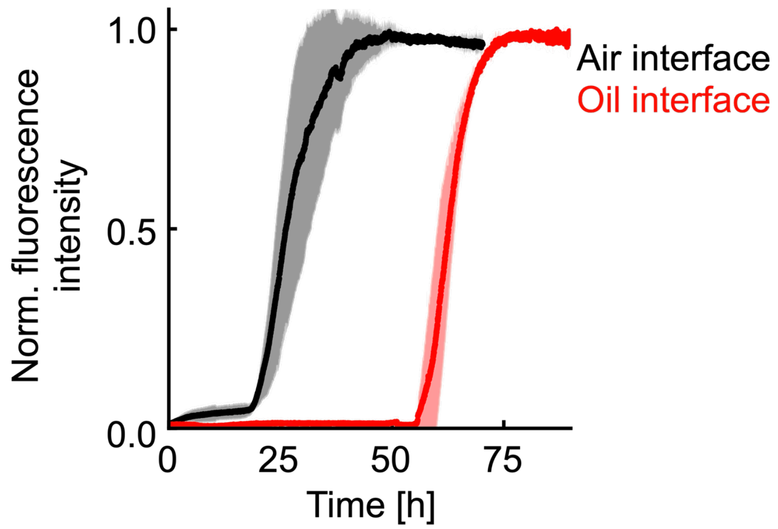

Figure S17: Normalized fluorescence intensity over time to follow the fiber formation under standard conditions (5 mM Ac-(FY(OMe)RCG)$_2$D-OH, 200 mM MES buffer at pH = 5.3, 2.5 µM ThT) with an air-solution interface (black) and an oil-solution interface (red). In between the measurements, the plate was shaken. Error bars are from triplicates (N = 3).

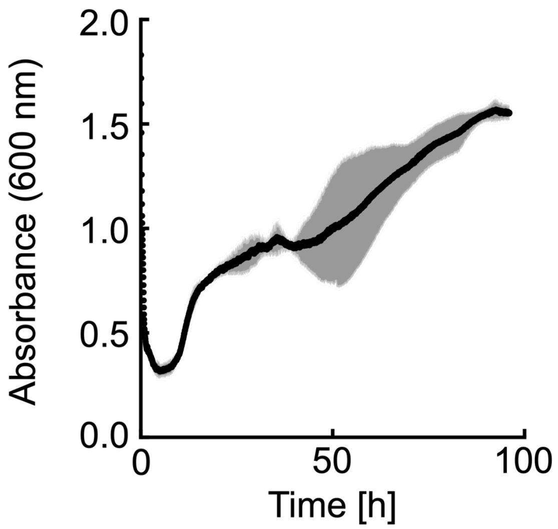

Figure S18: Absorbance over time under standard conditions (5 mM Ac-(FY(OMe)RCG)$_2$D-OH, 200 mM MES buffer at pH = 5.3, 25 mM EDC) topped with 1M DIC containing mineral oil. Error bars are from triplicates (N = 3).

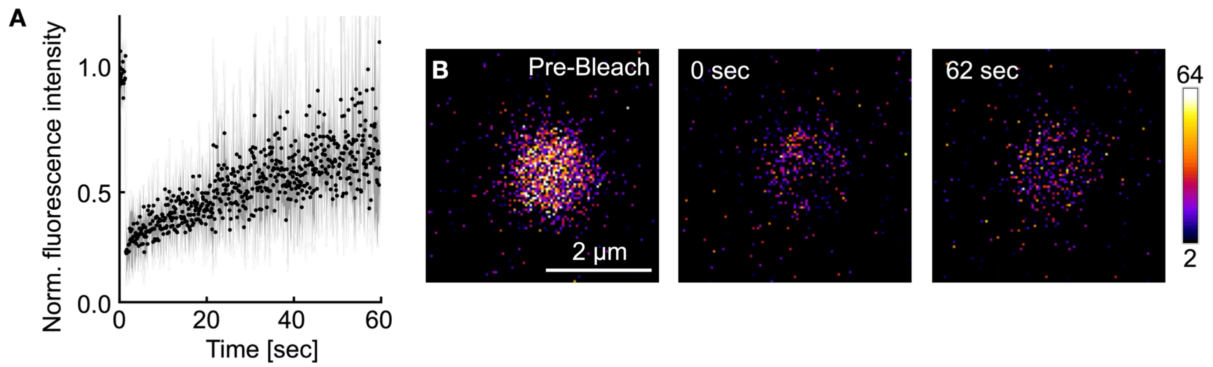

Figure S19: FRAP of an active, simple coacervate droplet that was sustained for three days. A) Normalized fluorescence intensity over time after photobleaching of simple coacervates (5 mM Ac-(FY(OMe)RCG)$_2$D-OH, 200 mM MES buffer at pH = 5.3) after fueling for three days. Error bars are from triplicates (N = 3). **B)** Confocal micrographs of the FRAP experiment shown in B. The scale bar is 2 μm.

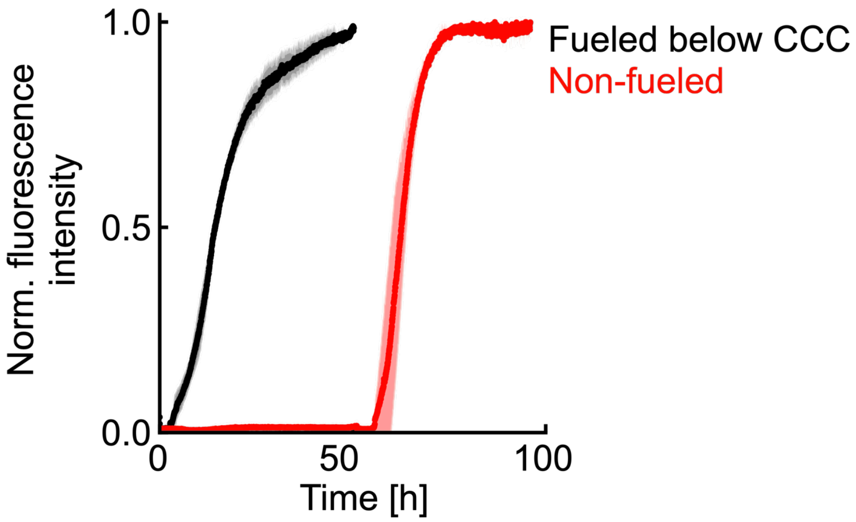

Figure S20: Normalized fluorescence intensity over time to follow the fiber formation under standard conditions (5 mM Ac-(FY(OMe)RCG)$_2$D-OH, 200 mM MES buffer at pH = 5.3, 2.5 μM ThT) with an oil-solution interface of a sample fueled with 5 mM EDC and 1 M DIC in the mineral oil (black) and a non-fueled sample (red). Error bars are from triplicates (N = 3).

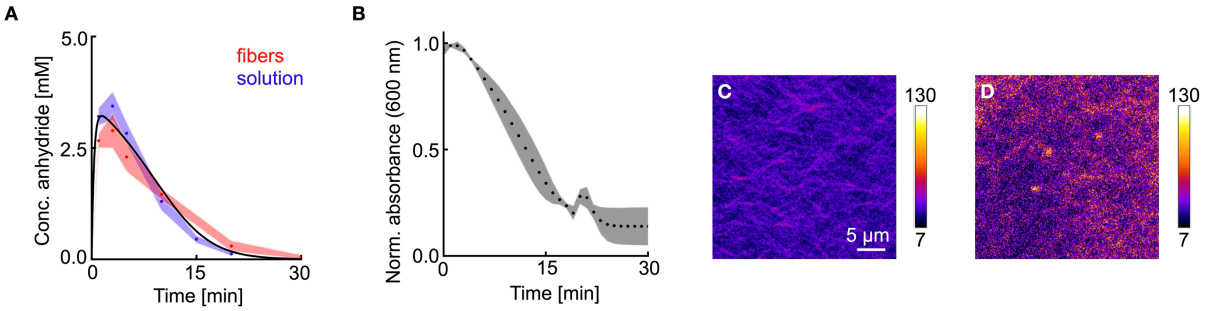

Figure S21: Fueling preformed fibers with EDC. For all experiments, fibers were formed using 5 mM Ac-(FY(OMe)RCG)$_2$D-OH, 200 mM MES buffer at pH = 5.3. **A**) Anhydride concentration over time of a fueled fresh solution (blue) and fibers (red) using 25 mM EDC measured by HPLC. The black solid line is the kinetic profile of the kinetic model. **B**) Normalized absorbance over time of fueled fibers using 25 mM EDC. **C**) Confocal micrograph of preformed fibers. The scale bar is 5 µm. **D**) Confocal micrograph after fueling the fibers shown in C with 25 mM EDC. The scale bar is 5 µm.

Supplemental references:

1. Poprawa, S.M., Stasi, M., Kriebisch, B.A.K., Wenisch, M., Sastre, J., and Boekhoven, J. (2024). Active droplets through enzyme-free, dynamic phosphorylation. Nat. Commun. *15*, 4204. https://doi.org/10.1038/s41467-024-48571-z.
2. Wenisch, M., Li, Y., Braun, M.G., Eylert, L., Späth, F., Poprawa, S.M., Rieger, B., Synatschke, C.V., Niederholtmeyer, H., and Boekhoven, J. (2025). Toward synthetic life—Emergence, growth, creation of offspring, decay, and rescue of fuel-dependent synthetic cells. Chem *11*, 102578. https://doi.org/10.1016/j.chempr.2025.102578.
3. Späth, F., Donau, C., Bergmann, A.M., Kränzlein, M., Synatschke, C.V., Rieger, B., and Boekhoven, J. (2021). Molecular Design of Chemically Fueled Peptide–Polyelectrolyte Coacervate-Based Assemblies. J. Am. Chem. Soc. *143*, 4782–4789. https://doi.org/10.1021/jacs.1c01148.
4. Bergmann, A.M., Bauermann, J., Bartolucci, G., Donau, C., Stasi, M., Holtmannspötter, A.-L., Jülicher, F., Weber, C.A., and Boekhoven, J. (2023). Liquid spherical shells are a non-equilibrium steady state of active droplets. Nat. Commun. *14*, 6552. https://doi.org/10.1038/s41467-023-42344-w.
5. Spoelstra, W.K., Van Der Sluis, E.O., Dogterom, M., and Reese, L. (2020). Nonspherical Coacervate Shapes in an Enzyme-Driven Active System. Langmuir *36*, 1956–1964. https://doi.org/10.1021/acs.langmuir.9b02719.
6. Kang, M., Day, C.A., Kenworthy, A.K., and DiBenedetto, E. (2012). Simplified equation to extract diffusion coefficients from confocal FRAP data. Traffic Cph. Den. *13*, 1589–1600. https://doi.org/10.1111/tra.12008.
7. CurveFitter (ImageJ API) https://imagej.net/ij/developer/api/ij/ij/measure/CurveFitter.html.
8. Panda, C., Sharma, L.G., and Pandey, L.M. (2023). Experimental procedures to investigate fibrillation of proteins. MethodsX *11*, 102445. https://doi.org/10.1016/j.mex.2023.102445.
9. Waeytens, J., Mathurin, J., Deniset-Besseau, A., Arluison, V., Bousset, L., Rezaei, H., Raussens, V., and Dazzi, A. (2021). Probing amyloid fibril secondary structures by infrared nanospectroscopy: experimental and theoretical considerations. Analyst *146*, 132–145. https://doi.org/10.1039/D0AN01545H.
10. Highly accurate protein structure prediction with AlphaFold | Nature https://www.nature.com/articles/s41586-021-03819-2.
11. Meisl, G., Kirkegaard, J.B., Arosio, P., Michaels, T.C.T., Vendruscolo, M., Dobson, C.M., Linse, S., and Knowles, T.P.J. (2016). Molecular mechanisms of protein aggregation

from global fitting of kinetic models. Nat. Protoc. *11*, 252–272. https://doi.org/10.1038/nprot.2016.010.
